# Performance of Microsoft Copilot in the Diagnostic Process of Pulmonary Embolism

**DOI:** 10.5811/westjem.24995

**Published:** 2025-07-13

**Authors:** Banu Arslan, Mehmet Necmeddin Sutasir, Ertugrul Altinbilek

**Affiliations:** Ministry of Health Sisli Hamidiye Etfal Training and Research Hospital, Department of Emergency Medicine, Istanbul, Türkiye

## Abstract

**Introduction:**

Patients with pulmonary embolism (PE) often present with non-specific signs and symptoms mimicking other conditions and complicating diagnosis. In this study we aimed to evaluate the performance of an artificial-intelligence tool, Microsoft Copilot, in the diagnostic process of PE, using clinical data including demographics, complaints, and vital signs.

**Methods:**

We conducted this study using 140 clinical vignettes, including 70 patients with and 70 patients without PE. The vignettes were derived from published case reports within the last 10 years. We used Copilot for its free GPT-4 integration to analyze clinical data and answer two questions after each vignette. We compared Copilot’s ability to identify PE within the top 10 differential diagnoses, and its ability to predict the risk of PE when compared to the use of the Wells score by two independent investigators.

**Results:**

Copilot correctly included PE in the differential diagnosis in 94.3% of cases by listing it within the top 10 conditions. Risk assessment by Copilot yielded significantly higher levels in patients with PE (*P*<.05). No statistically significant difference was found in the Wells scores between patients with PE and without PE (*P*>.05). Copilot demonstrated better discriminatory power than the Wells score in risk assessment of PE (area under the curve 0.713 vs 0.583), with statistical significance (*P*<0.001 vs *P*=.091). Sensitivity, specificity, positive predictive value, and negative predictive value for discriminating between the combination of low- and intermediate- vs high-risk categories were 34%, 97.1%, 92.3%, and 59.6%, respectively.

**Conclusion:**

This study explores the potential of Copilot as a tool in clinical decision-making, demonstrating a high rate of correctly identifying PE and improved performance over the Wells score. However, further validation in larger populations and real-world settings is crucial to fully realize its potential.

## INTRODUCTION

Pulmonary embolism (PE) is a life-threatening medical emergency. It occurs when a thrombus blocks the pulmonary artery or its branches. After heart attack and stroke, it is the third most common acute cardiovascular syndrome.^1^ A timely diagnosis is crucial because PE is associated with high mortality rates. If left untreated, 30% of PE patients, whereas 8% die after timely treatment.^2^

The diagnosis of PE is challenging due to a range of issues from subtle and non-specific symptoms to the complexities of medical imaging interpretation. The diagnosis of PE heavily relies on a computer tomography pulmonary angiography (CTPA) for its high sensitivity and specificity.^3^ However, this imaging technique is costly and time-consuming, and requires radiologists’ expertise. Furthermore, it carries some risks that might primarily affect patient safety such as ionizing radiation exposure, anaphylactic response to contrast agent, contrast-induced nephropathy, dye extravasation, and incidental findings causing unnecessary procedures.^4^,^5^ To avoid overuse of CTPA, current guidelines recommend using a pre-test probability approach by using empirical clinical assessment or standardized clinical prediction rules for hemodynamically stable patients.^6^ However, these widely accepted traditional scoring systems often lack the desired accuracy. Additionally, adherence to these risk scores is still low.^7^

As the healthcare landscape continues to evolve, there is a growing recognition of advanced technologies in the diagnostic process of PE. A potential approach to improve diagnosis could be the utilization of advanced methods for clinical data analysis. By leveraging machine-learning algorithms, artificial intelligence (AI) can analyze vast amounts of clinical data and identify patterns that may indicate the presence of PE. Recent studies indicate that AI demonstrates proficiency in detecting PE while extending its capabilities to risk assessment, risk stratification, and even mortality prediction.^8^–^11^

The exploration of potential applications of large language models (LLM) within the medical field has gained momentum over the past year. Notably, the introduction of ChatGPT (OpenAI, San Francisco, CA), Bard (Google LLC, Mountain View, CA) and Copilot (Microsoft Corporation, Redmond, WA) stands out as a significant contributor to this trend. Recent studies have demonstrated the multifaceted capabilities of these LLMs in several medical domains, including streamlining clinical workflows,^12^ contributing to personalized learning,^13^ conducting comprehensive literature reviews, and providing up-to-date medical information.^14^ Furthermore, these LLMs have shown high accuracy by generating differential diagnosis lists based on provided clinical vignettes.^15^,^16^

A study by Hirosawa et al demonstrated that ChatGPT-4 correctly identified PE in 83% of cases when included in the top 10 differential diagnoses.^16^ However, Hirosawa and colleagues analyzed a broad range of diseases using a relatively small sample size of 52 cases from a single department, limiting its generalizability. Given the potential of AI tools to aid in identifying PE, further research is needed to assess their performance in a more focused and systematically controlled setting. In this study, we evaluated the effectiveness of an LLM-based generative AI tool in improving the diagnostic process and enhancing the estimation of pre-test probability for PE using previously published case reports. We do not propose Copilot as a definitive diagnostic tool or a replacement for CTPA. Instead, we evaluate how AI can assist in determining which patients would benefit most from further diagnostic testing, potentially optimizing resource use and improving patient outcomes.

Concerns have been raised regarding the accuracy of LLMs in providing scientific answers during emergency situations. A recent study by Yau et al highlighted significant deficiencies in free versions of ChatGPT, Bard, Bing, and Claude AI, including inadequate medical and scientific accuracy, incomplete information, dissemination of dangerous information, and absence of source citations.^17^ However, to date there has been no study conducted to evaluate the capability of these LLMs in diagnosing specific medical emergencies.

**Population Health Research Capsule**

What do we already know about this issue?

*Diagnosing PE is challenging due to the nonspecific nature of its symptoms and the complexities associated with imaging. AI and LLMs show promise but face accuracy concerns*.

What was the research question?

*Can Copilot aid PE diagnosis by generating accurate differential diagnosis list and estimating pre-test probability using clinical data*.

What was the major finding of the study?

*Copilot identified PE in top 10 diagnoses with 94.3% accuracy and demonstrated a higher area-under-the-curve (AUC) for the receiver operating characteristic (ROC) curve than Wells score (0.713 vs. 0.583)*.

How does this improve population health?

*By improving the diagnostic process of PE, Copilot may be able to optimize resource use, reduce unnecessary tests, and enhance patient outcomes, which may contribute to better population health*..

In this study, we used the free version of Microsoft Copilot, a widely used but under-represented AI model in the medical literature. By leveraging Copilot, we introduce a novel approach that mirrors how clinicians construct differential diagnoses and assess PE risk levels. At the start of the study, Copilot offered several advantages over OpenAI’s ChatGPT. For example, Copilot provides three different chat tones: creative; balanced; and precise. The precise mode is designed to provide concise, search-focused answers. On the other end of the spectrum, the creative mode generates responses that are more elaborate and descriptive. The balanced mode, as the name suggests, strikes a balance between the two, offering responses that are neither too brief nor too detailed.^18^ Since December 2023, Microsoft Copilot has been available on the Android operating system, which provides direct access to the chatbot. Additionally, it allows using GPT-4 with the “use GPT-4” button at no cost.

## MATERIALS AND METHODS

### Definition of Outcomes

The primary outcome of this study was the ability of Microsoft Copilot to accurately identify PE based on clinical data. We assessed the performance of Copilot in listing PE within the top 10 differential diagnosis list. Including 10 possible diagnoses allowed us to better assess Copilot’s “diagnostic” capabilities, evaluate its performance in complex cases, and ensure comparability with existing literature.

The secondary outcome was to assess the ability of Copilot to accurately determine the risk of PE. The Wells score, also known as the Wells criteria, is widely used to determine the risk of PE.^19^ This scoring system helps healthcare professionals estimate the probability of PE based on various clinical factors. The Wells score incorporates both clinical signs and symptoms to stratify patients into low, moderate, or high probability categories (Table 1).^20^ By assigning points to different criteria, the Wells score aids in guiding further diagnostic testing and determining the most appropriate management strategies for individuals. In this study, the Wells score was independently calculated by two investigators based on review of clinical vignettes. These investigators were not blinded to the full text of the case reports, which may have introduced bias given the likely knowledge of the true diagnosis in calculating the “most likely diagnosis” component of the Wells score. Discrepancies between the two investigators were resolved through discussions. The risk assessment was conducted through Microsoft Copilot by asking: “Can you rate the risk of PE in this case as low risk, intermediate risk, or high risk?”

### Study Design

We conducted this study using clinical vignettes derived from published case reports. To be enrolled in the study, case reports had to meet the following criteria: 1) published in the last 10 years; 2) written in the English language; 3) involved adult patients (≥ 18 years) and available in full text at no cost; 4) provided key clinical details for crafting clinical vignettes; 5) involved patients with suspected PE; and 6) confirmed or excluded PE through CTPA. We excluded case reports that had incomplete diagnostic information, inconclusive CTPA results, or described embolization of other materials into the pulmonary circulation. We also excluded case reports involving postmortem diagnoses or patients who had already been hospitalized for more than three days.

The study was conducted in accordance with the Standards for Reporting Diagnostic Accuracy (STARD) guidelines for reporting diagnostic accuracy. Approval by the ethics committee was not obtained, since the study only used case vignettes from published case reports.

### Literature Search and Case Report Selection

In September 2023, two authors conducted a systematic search on the PubMed database using the search terms “((pulmonary embolism) AND (acute)) OR ((dyspnea) AND (acute)).” By selecting “case report” as the article type, a total of 7,611 case reports were retrieved. Following additional filters, including “adult,” “English,” “10 years,” and “free full text,” 1,184 case reports were selected for the initial assessment.

**Figure 1:**
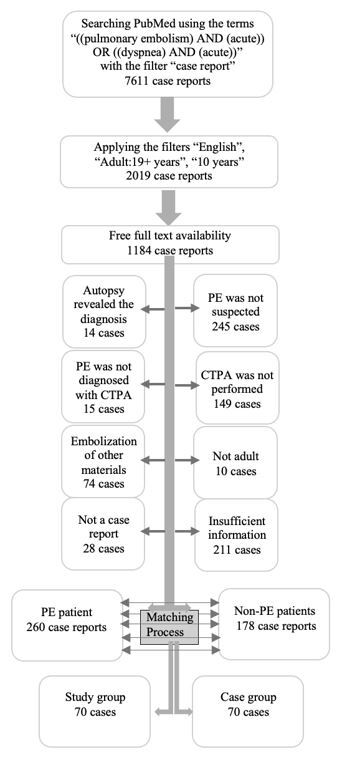
Case selection process in a study of the ability of Microsoft Copilot to aid in the diagnostic process for pulmonary embolism. *PE*, pulmonary embolism, *CTPA*, computed tomography pulmonary angiography.

First, we assessed titles and abstracts to exclude cases that were clearly irrelevant. Then, we reviewed full-text reports. Based on the inclusion and exclusion criteria, we identified 438 case reports for patients with suspected PE who underwent CTPA. Based on CTPA results, the patients were divided into two groups: those with PE (study group, 260 cases) and those without (control group, 178 cases). Using a manual exact-matching process, each PE patient was paired with a non-PE patient whose age was within ±1 year and whose sex matched. Following meticulous evaluation and matching, we divided 140 of these case reports (70 in the study group and 70 control group), which represented the maximum number of cases that qualified for inclusion after applying our case selection and matching criteria. The case selection process is visually presented in Figure 1.

### Clinical Vignettes

In October 2023, one of the authors extracted the data from the selected case reports and displayed them as detailed clinical vignettes (Appendix). Each clinical vignette was written in English and included the following: age; sex; chief complaint; chronic medical conditions; history of present illness; vital parameters; and physical examination findings. The results of routine laboratory tests, imaging studies, differential diagnoses, final diagnosis, titles, figures, legends, and tables were removed and not included in the clinical vignettes.

In December 2023, we submitted each clinical vignette to Microsoft Copilot. After inputting the clinical vignette, the following questions were prompted, and the answers were recorded:

1. Can you list 10 possible diagnoses for the clinical vignettes above, ordered from most likely to least likely?
2. Can you rate the risk of PE in this case as low risk, intermediate risk, or high risk?

**Figure 2:**
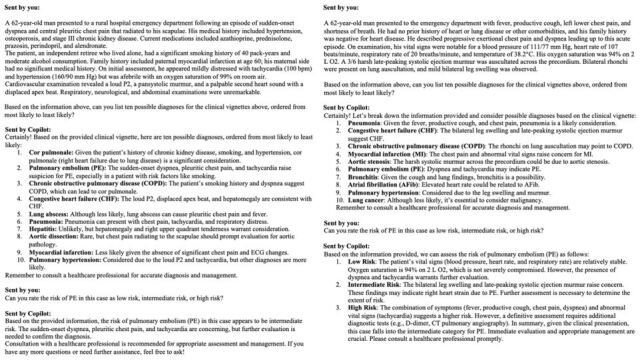
Example of the rankings generated by Copilot for two different patients. Both patients were 62-year-old males admitted to an emergency department. The first patient was diagnosed with pulmonary embolism,^21^ and the second was diagnosed with acute viral bronchitis, severe bicuspid aortic valve stenosis, and coronary-pulmonary artery fistulas.^22^

By employing this two-step process, we introduced a novel approach that mirrors the real-world diagnostic workflow commonly used in emergency departments, aiming to enhance the diagnostic process. An example of conversation between one of the authors and Copilot is shown in Figure 2. After recording the answers, to prevent any potential interference from prior responses, we cleared the conversation every time before introducing new clinical vignette.

### Microsoft Copilot

Microsoft Copilot is an AI-powered productivity tool designed to assist users in various tasks. It employs a combination of LLMs, a type of AI algorithm that uses deep-learning methods and extensive data sets to comprehend, summarize, predict, and generate content.^23^ It runs on pre-trained models such as Generative Pre-trained Transformer-4 (GPT-4) to excel in these tasks. While GPT-4 is considered to be the most advanced language model,^24^ details of its architecture are not publicly available. For this study, we chose the “precise” mode to ensure concise and focused answers.

### Statistical Analysis

We performed statistical analyses using SPSS software v28.0 (SPSS Statistics, IBM Corp, Armonk, NY). Descriptive statistics, including means, standard deviations, medians, minimum, maximum values, frequencies, and percentages were reported. To evaluate the normality of the distribution of continuous variables, we applied the Shapiro-Wilk test. For normally distributed data, the independent samples *t*-test was used to compare the means between two independent groups. We used the Mann-Whitney U test to compare the medians of ages, and the chi-square test to compare the distribution of categorical variables such as the proportions of male and female patients, Copilot’s risk assessment, and the Wells score risk assessment between two groups. The receiver operating characteristic (ROC) analysis was conducted to evaluate the performance of variables. This involved calculating the area under the ROC curve (AUC), which provides a measure of how well a parameter can distinguish between two diagnostic groups (eg, PE vs non-PE). The AUC values range from 0.5 to 1. An AUC of 0.5 indicates no discrimination ability, while values below 0.7 suggest poor performance. An AUC value greater than 0.7 reflects a reasonably good model, and an AUC of 1 denotes perfect discrimination.

## RESULTS

We conducted this study with a total of 140 clinical vignettes, including 70 patients with PE (study group) and 70 patients without PE (control group). Ages of the patients ranged from 21–86 years with a mean of 54 ± 16.3. Most patients in each group were female (54.3%). We were unable to detect a statistically significant differences between the two groups by age (*P* = .96) and sex (*P* = 1.00) by design. Dyspnea was the most frequent presenting symptom among all patients, but notably more prevalent in those without PE (92.8% v 70%). Symptoms such as chest pain and syncope were more prevalent in patients with PE, occurring in 47.1% and 15.7% of cases, respectively, compared to 21.4% and 1.4% in those without PE. Treatment patterns also differed between groups, with a higher percentage of PE patients (78.6%) receiving medical treatment compared to those without PE (58.6%). Regarding outcomes, most patients were discharged, but PE patients were more likely to be admitted to the intensive care unit (ICU) than those without PE (10% v 1.4%). The study population is described in detail in Table 2. Microsoft Copilot correctly listed PE in the top 10 differentials for 66/70 (94.3%) cases in the study group and 58/70 (82.9%) in the control group. Patients with PE were 3.41 times more likely to have PE in the top 10 differential diagnoses compared to patients without PE (odds ratio 3.41; 95% confidence interval [CI] 1.04–11.17) (Table 3).

The risk of PE was assessed based on the distributions of patients across different risk categories (“high,” “intermediate,” and “low”), as determined by Copilot and the Wells scores. Copilot classified patients without PE as follows: 2 patients (2.9%) as high risk; 28 patients (40%) as intermediate risk; and 40 patients (57.1%) as low risk. Patients with confirmed PE were also classified by Copilot as follows: 24 patients (34.3%) as high risk; 27 patients (38.6%) as intermediate risk; and 19 patients (27.1%) as low risk. The level of risk determined by Copilot was significantly higher in the study group than the control group (*P* < .05). Remarkably, we were unable to detect a statistically significant difference in the Wells scores between the groups with and without PE (*P* > .05) (Table 4).

**Figure 3:**
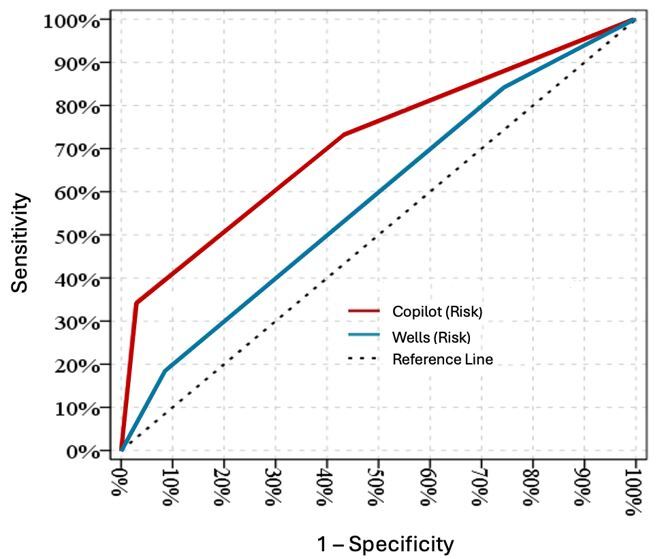
The areas under the curve of the receiver operating characteristic for Copilot and the Wells score.

We evaluated the performance of Copilot and the Wells score in risk assessment for PE through AUC analysis. The AUC was 0.713 (Cl 95% 0.628 – 0.798) for Copilot. The Wells score had a lower AUC (0.583, Cl 95% 0.489 – 0.677) that was not statistically significant (*P* = .09), indicating poor performance in the risk assessment (Table 5). The AUCs of the ROC curves for Copilot and the Wells score are shown in the same figure (Figure 3). The agreement between these tools was 47.9% with a Cohen kappa of 0.26 (Table 6).We calculated the sensitivity, specificity, positive predictive value (PPV), and negative predictive value (NPV) of Copilot based on risk levels (Table 7). For the discrimination between low + intermediate and high-risk categories, the sensitivity, specificity, PPV, and NPV were 34%, 97.1%, 92.3%, and 59.6%, respectively. Conversely, for the discrimination between low and intermediate + high-risk categories, these values were 72.9%, 63%, 57.1%, and 67.8%, respectively.

## DISCUSSION

The current study involved a total of 140 clinical vignettes presenting patients with suspected PE. Patients were equally distributed into two groups according to the CTPA results: patients with and without PE. The evaluation was based on clinical data including age, sex, chief complaint, medical history, vital parameters, and physical examination findings. The demographic distribution in terms of age and sex was statistically similar in both groups (*P* < .05). We employed a two-step process to generate and prioritize differential diagnoses and determine risk levels for PE. Our findings underscore the remarkable performance of Copilot in generating differential diagnosis lists and demonstrating better capabilities in predicting the risk of PE compared to the Wells score, especially in complex clinical scenarios.

The literature has investigated the effectiveness of AI in diagnosing PE and recognizing patients at high risk. By analyzing vast clinical data and identifying patterns and trends within this information, AI offers invaluable support to clinicians and augments the precision of medical diagnoses. For example, Rucco et al used data on 28 variables such as age, previous deep vein thrombosis (DVT), and hemoptysis of 1,427 patients with suspected PE. Their neural hypernetwork correctly recognized 94% of the PE cases.^25^ In a separate study, Ryan et al tested three distinct machine-learning (ML) models on clinical data extracted from electronic health records of 60,297 patients and demonstrated the capability of ML-based models to identify patients at high risk for developing PE.^26^

Copilot leverages a combination of advanced algorithms, LLMs and ML techniques to comprehend user input and generate appropriate responses. We hypothesized that Copilot’s ML algorithms could be configured to analyze extensive clinical data, including patient demographics, medical history, and symptoms to identify patterns indicative of PE, similar to the approaches undertaken by Rucco et al and Ryan et al.^25^,^26^ With this study, we aimed to evaluate the potential of Copilot in two specific tasks: to enhance the diagnostic process with generating accurate differential diagnosis lists; and to improve the estimation of pretest probability for PE with an ultimate goal to determine whether AI can support and improve clinical decision-making and workflow in emergency medicine.

Copilot also included PE in the differential diagnosis for 82.9% of the control group cases. This may raise questions about the tool’s reliability and the potential randomness of these occurrences. However, it is crucial to consider that case reports published in the medical literature often involve highly complex scenarios, featuring rare presentations or unique clinical settings. Likewise, the clinical vignettes included in our study mostly fell into the intermediate-high risk group risk (84.3% in the study group and 74.3% in the control group). This emphasizes the challenging nature of the task undertaken by Copilot. However, even within this highly complex environment, our comparative analysis revealed a consistent trend: Copilot consistently listed the correct diagnosis near the top diagnosis in the study group (*P* < .05). This suggests that Copilot can prioritize PE based on the presented symptoms and patient history and could serve as valuable clinical decision-support tools, providing meaningful assistance to clinicians.

As the second task, Copilot was directed to predict the risk of PE for each vignette. Notably, the risk (low-intermediate-high) determined by Copilot was significantly higher in patients with confirmed PE. On the other hand, a statistically significant difference in the Wells scores between the groups was not detected. The Wells score is a widely accepted clinical prediction tool used to classify patients suspected of having PE into low, intermediate, or high-risk groups. This classification aids clinicians in selecting the next investigative step, such as D-dimer testing, CTPA, or lung scintigraphy. Even though the Wells score is an essential part of determining the likelihood of PE, its accuracy in certain patient populations has been subject to scrutiny.^27^,^28^ Girardi et al reported that the Wells score is unreliable for predicting PE in ICU patients.^29^ Contrary to the reported incidence rate of 1.3% for the low-risk group,^30^ they detected PE in 26.8% of patients classified as low probability by the Wells score. This discrepancy may be due to the fact that factors associated with high risk for PE may be more prevalent in ICU patients and clinical prediction tools may exhibit varied performance depending on the settings. Similarly, our study found that 15.7% of patients categorized as low risk by the Wells score had PE.

The AUC values observed in our study (Copilot=0.713 vs Wells=0.583) may suggest that Copilot demonstrated better discriminatory ability than the Wells score for stratifying PE risk, particularly in complex cases. Several factors may contribute to Copilot’s better performance. First, Copilot, with its advanced AI foundation, was able to process large amounts of patient data and assess a broader array of variables and their interactions. On the other hand, the Wells score relies on a limited set of predefined criteria, such as history of DVT, surgery, or cancer. This broader analysis may lead to a more nuanced understanding of individual patient risk, particularly in cases with complex or ambiguous presentations. These findings suggest that Copilot may be more sensitive in identifying patients who are truly at higher risk for PE, allowing for better triage decisions and timely interventions. However, validation with larger populations is warranted in future studies.

## LIMITATIONS

Regarding the limitations of this study, it is crucial to acknowledge the potential impact of publication bias in the included case reports. Our data rely on clinical vignettes derived from case reports. There is a tendency for case reports with positive outcomes to be preferentially published, thereby introducing a potential source of bias. Additionally, written clinical vignettes may not fully represent the breadth of presentations encountered in actual clinical practice, and omit the general appearance of the patients, which could affect medical decision-making. Real-world emergency departments see a mix of low, moderate, and high-risk patients, while published cases may disproportionately feature high-risk or ambiguous cases. However, our findings deviate from the reported incidence rates in terms of severity of the disease. In our study, PE was present in 15.7% of the low-risk group, 65.7% in the intermediate-risk group, and 18.6% in the high-risk group according to the Wells score, whereas Ceriani et al reported lower rates of 6%, 23%, and 49%, respectively, in their meta-analysis.^31^ This disparity may result from the fact that published cases often involve unique or rare aspects of a disease, unusual presentations, or rare complications.

Over-representation of complex cases can introduce a form of selection bias. These limitations affect the generalizability of our study by potentially skewing the results toward more complex or atypical cases, which may not accurately reflect the broader population of patients encountered in routine emergency settings. Another potential limitation is the possibility that Copilot may have been previously exposed to some of the clinical vignettes used during its training, as they were sourced from publicly available materials. This could have influenced the model’s performance and limit the generalizability of the findings. Also, case matching was performed based only on age and sex, which are not strong predictors of PE risk. Other potential confounding factors, such as comorbidities and clinical presentation, were not controlled for, which may have led to baseline differences between the groups. The investigators who adjudicated the Wells score were not blinded to the full text of the vignette, which may have introduced bias, particularly in assessing whether PE was the most likely diagnosis. Lastly, the probabilistic nature of LLMs introduces variability in outputs, which may affect reproducibility. This inherent characteristic, along with potential updates to the model, can lead to differences in results even when using the same clinical vignettes, limiting the consistency of findings over time.

## CONCLUSION

This study suggests that Microsoft Copilot may have potential in generating differential diagnoses and assisting in risk prediction for patients with suspected pulmonary embolism. Copilot identified PE within the top 10 differential diagnoses with 94.3% accuracy and demonstrated a higher AUC than the Wells score in risk stratification (0.713 vs 0.583). Additionally, Copilot accurately identified PE near the top diagnosis in the study group, indicating its possible utility as a clinical decision-support tool. By integrating with electronic health records and analyzing extensive clinical data, Copilot can serve as a real-time decision-support tool for clinicians. It can offer recommendations based on the patient’s history, symptoms, and other relevant data, aiding in more informed decision-making. Additionally, its ability to stratify PE risk into low, intermediate, and high categories could contribute to more tailored patient management strategies with individual risk levels. However, this study serves as a preliminary analysis of Copilot’s feasibility. Further validation with larger populations and real-world clinical settings is essential to confirm its efficacy and reliability.

## Supplementary Information



## Figures and Tables

**Table 1:** Wells score for pulmonary embolism.

Characteristics		Points
Clinical signs and symptoms of DVT		3
An alternate diagnosis is less likely than PE		3
Heart rate >100 beats/minute		1.5
Immobilization or surgery in the previous 4 weeks		1.5
Previous DVT / PE		1.5
Hemoptysis		1
Malignancy (treatment currently, in the previous 6 months, or palliative)		1

Risk	Points required	Risk of PE

Low	0–1	3.6%
Moderate	2–6	20.5%
High	>6	66.7%

*DVT*, deep vein thrombosis*; PE*, pulmonary embolism.

**Table 2:** Demographic and clinical characteristics of the population in a study of the ability of Microsoft Copilot to aid in the diagnostic process for pulmonary embolism. (n=140).

		Pulmonary embolism (−) (n=70)					Pulmonary embolism (+) (n=70)					
	
		Mean±SD n-%			Median	I.Q-3.Q	Mean±SD n-%			Median	I.Q-3.Q	*P-*value
Age		54	±	16.3	57.0	43.8–63.5	53.9	±	16.4	57.0	43.8–63.5	0.955^m^
Sex	Female	38		54.3			38		54.3			1.000x^2^
	Male	32		45.7			32		45.7			
Chief complaint	Dyspnea	65		92.8			49		70			
	Cough	18		25.7			14		20			
	Chest pain	15		21.4			33		47.1			
	Fever	5		7.1			10		14.3			
	Leg pain	4		5.7			9		12.9			
	Hemoptysis	3		4.3			3		4.3			
	Syncope	1		1.4			11		15.7			
Chronic medical condition	HT	24		34.3			20		28.6			
	Cancer	12		17.1			6		8.6			
	DM	9		12.9			5		7.1			
	HL	7		10			5		7.1			
	Asthma	5		7.1			2		2.9			
	COPD	5		7.1			4		5.7			
	Obesity	5		7.1			9		12.9			
	PE	4		5.7			6		8.6			
	DVT	3		4.3			5		7.1			
	None	19		27.1			26		37.1			
Treatment	Medical	41		58.6			55		78.6			
	NSI	15		21.4			8		11.4			
	Surgery	14		20			7		10			
Outcome	Discharged	59		84.3			59		84.3			
	ICU	1		1.4			7		10			
	Deceased	10		14.3			4		5.7			

m , Mann-Whitney U test;

X^2^, chi-square test

*HT*, hypertension; *DM*, diabetes mellitus; *HL*, hyperlipidemia; *COPD*, chronic obstructive pulmonary disease; *PE*, pulmonary embolism*; DVT*, deep vein thrombosis; *NSI*, non-surgical intervention; *ICU*, intensive care unit.

**Table 3:** Ranking of pulmonary embolism (PE) in a differential diagnosis list, along with the distribution of cases with PE and without PE.

	Rank in the list	Pulmonary embolism (−)			Pulmonary embolism (+)			
	
		(n)	(%)	Cumulative (%)	(n)	(%)	Cumulative (%)	*P*-value
**Microsoft Copilot**	1	2	2.9%	2.9 %	22	31.4%	31.4 %	.034*
	2	8	11.4%	14.3%	14	20.0%	51.4%	
	3	10	14.3%	28.6%	9	12.9%	64.3%	
	4	16	22.9%	51.5%	9	12.9%	77.2%	
	5	10	14.3%	65.8%	5	7.1%	84.3%	
	6	6	8.6%	74.4%	1	1.4%	85.7%	
	7	2	2.9%	77.3%	1	1.4%	87.1%	
	8	1	1.4%	78.7%	2	2.9%	90%	
	9	3	4.3%	83%	2	2.9%	92.9%	
	10	0	0.0%	83%	1	1.4%	94.3%	
	≥11	12	17.1%	100%	4	5.7%	100%	

* Chi-square test.

**Table 4:** Comparison of risk assessment between patients with pulmonary embolism and without pulmonary embolism.

	Pulmonary embolism (−)		Pulmonary embolism (+)		
	
	N	%	n	%	*P* **(**x^2^)
***Copilot***					
Low	40	57.1%	19	27.1%	< .001
Intermediate	28	40.0%	27	38.6%	
High	2	2.9%	24	34.3%	
***Wells Score***					
Low	18	25.7%	11	15.7%	**.118**
Intermediate	46	65.7%	46	65.7%	
High	6	8.6%	13	18.6%	

X^2^, chi-square test.

**Table 5:** Data on area under the curve in a study of the ability of Microsoft Copilot to aid in the diagnostic process for pulmonary embolism.

	Area under curve	95% Confidence interval	P-value
Copilot	0.713	0.628 – 0.798	< .001
Wells score	0.583	0.489–0.677	.091

**Table 6:** Agreement between the Wells score and Copilot for the classification of all patients into the three risk levels (low, intermediate and high) in a comparison study of the ability of Copilot and Wells score to risk-stratify patients from published case reports into risk categories for pulmonary embolism.

		Pretest probability using Copilot			Rapport	*P*/kappa
		Low	Intermediate	High		
Pretest probability using the Wells score	Low	20	9	0	47.9%	***.002/0.26***
	Intermediate	37	38	17		
	High	2	8	9		

**Table 7:** Sensitivity, specificity, positive predictive value, and negative predictive value for discriminating patients with and without pulmonary emboliism based on different risk categories.

	Pulmonary embolism (−)	Pulmonary embolism (+)	Sensitivity	PPV	Specificity	NPV	*P*
***Copilot***							
Low+Intermediate	68	46	34.3%	92.3%	97.1%	59.6%	< .001
High	2	24					
Low	40	19	72.9%	63.0%	57.1%	67.8%	*.001*
Intermediate+High	30	51					

*PPV*, positive predictive value; *NPV*, negative predictive value.
