# Supplementary material for: Performance of Microsoft Copilot in the Diagnostic Process of Pulmonary Embolism
Source: West J Emerg Med. 2025 Jul 13;26(4):1030–9. doi: 10.5811/westjem.24995 (PMC12342421; doi:10.5811/westjem.24995)
Supplement: Supplementary file 1 [file wjem-26-1030-s001.docx]

# Massive pulmonary embolism presenting with hemoptysis and S1Q3T3 ECG findings.

# A 61-year-old male patient was admitted to our hospital with chest pain, difficulty breathing, haemoptysis and general discomfort. His body mass index was 35, which is consistent with obesity, and he also had hypertension based on anamnesis. The patient was conscious, but there was a lack of cooperation and orientation. The Glasgow Coma Scale was 13 (eye = 6, motor = 4, verbal = 3), S1 (+) and S2 (+). His heart rate was 120/min, body temperature was 37 °C, oxygen saturation was 89%, systolic blood pressure was 90 mm/hg and diastolic blood pressure was 60mm/Hg. In addition to a positive Homan’s sign, there was swelling and erythema of the left leg.

# Pulmonary embolism mimicking acute myocardial infarction: a case report and review of literature

A 37-year-old woman, without past medical history, presented to emergency room in primary center, complaining of chest pain, acute coronary syndrome was suspected. Our emergency medical system received call for this patient and activated pre hospital emergency team for transfer. The patient suffered from continuous acute two hours before our intervention. She doesn't have previous history of similar episode. No previous history of coronary artery disease, peripheral vascular disease, stroke, malignancy, or venous thromboembolism was reported. There was no family history of thromboembolic disease. Physical examination revealed: a regular pulse rate 110 beats/min, blood pressure was 100/65mmHg, respiratory rate was 20 breaths/min, oxygen saturation was 95% at room air and 99% with 2l/min oxygen via nasal canula and temperature was 37°C. Cardiac auscultation was normal. There were no congestive neck veins.

# Aortic Arch Thrombus and Pulmonary Embolism in a COVID-19 Patient.

# An otherwise healthy 53-year-old woman presented with dyspnea, fever, and cough that had started approximately 10 days prior to her hospitalization. The patient tested positive for severe acute respiratory syndrome coronavirus 2 (SARS-CoV-2) infection. In the Emergency Department she was noted to be afebrile but hypoxic. Oxygen saturation was 84% on room air, improving to 88% with a nasal cannula and to 98% with a nonrebreather mask. No tachycardia is observed.

# Concomitant pulmonary embolism and upper limb ischaemia as a first presentation of a patent foramen ovale

# A 78-year-old female patient with Alzheimer’s dementia presented to the emergency department with syncope and dyspnoea. Initially measured saturation was 67%, and increased to 95% after using a non-rebreathing mask with 15 liters/minute of oxygen. The respiratory rate initially was 40 breaths/min, declining to 30 breaths/min with increasing saturation. Furthermore, the left arm was cold and peripheral pulsations were not palpable. There was a significant blood pressure difference between the right and the left arm, to the disadvantage of the left arm. Heart rate is 110 beats/min.

# Cold agglutinin disease and autoimmune hemolytic anemia with pulmonary embolism as a presentation of COVID-19 infection

the patient is a 51-year-old African American woman with a past medical history of right breast ductal carcinoma in situ diagnosed in 2012 status post lumpectomy, radiation and tamoxifen for 2 years. She was diagnosed with recurrent stage IA right breast cancer in 2019 and underwent a nipple-sparing mastectomy on January 16, 2020, with breast reconstruction on February 26, 2020. She also has a history of left lower extremity VTE in her 30 s provoked by oral contraceptive pills.

The patient presented to the emergency room on March 28, 2020, with fever, shortness of breath, malaise, rib, and back pain. In the emergency room, she was tachycardic with a heart rate of 112 beats/minute and febrile with 102.2°F. She had an oxygen saturation of 88% on room air.

# Concomitant coronary and pulmonary embolism associated with patent foramen ovale: A case report

A 59-year-old man was brought to the emergency department (ED) complaining of chest pain radiating to the throat and shortness of breath for 5 h. He had a previous history of hypertension, which was not well-controlled, and a 30 pack-year history of smoking. His heart rate was 84/min, respiratory rate was 24/min, oxygen saturation was 85% on room air, blood pressure was 114/85 mm Hg, and body temperature was 36.3°C in the ED. Physical examination revealed cyanotic lips, and clammy and cool extremities.

# Nitrous oxide inhalant abuse and massive pulmonary embolism in COVID-19

A 23-year-old male with a history of nitrous oxide abuse presented to the emergency department after being found face down Shortly thereafter, the patient developed worsened tachycardia of 139 beats per minute and blood pressure 73/57 mmHg, requiring vasopressor support. His oxygen saturation fell to 92%, with progressively cool and clammy extremities.

# What should be done in the event of simultaneous massive pulmonary embolism and myocardial infarction with ST elevation? (75).

An 85-year-old woman with no known prior diseases except hypertension/hypotension (HT) presented to emergency with dyspnea, chest pain and poor general condition. A brief syncope episode had occurred two hours prior to presentation. Upon physical examination, her blood pressure (BP) was 90/50 mmHg, pulse 130/min and respiratory rate 25 breaths/minute. She was found to have pitting edema and redness in the right calf and diffuse wheezing on physical examination.

The patient’s HR: 130/min.

# Spinal cord infarction secondary to pulmonary embolism-induced cardiac arrest: a case report

A 72-year-old woman without a history of any coexisting disease was admitted to the emergency room with a complaint of dyspnea and chest tightness. Physical examination showed obvious cyanosis of lips; vital signs indicated an unstable hemodynamic status, with a heart rate of 102 beats/min, a respiratory rate of 25 beats/min, and blood pressure of 88/51 mmHg, finger oxygen saturation of 61%, and body temperature of 36.0 °C. The initial electrocardiogram at admission suggested sinus tachycardia. CA occurred 10 minutes later, and high-quality CPR was performed immediately. can you give me ten possible differential diagnoses, ordered from most likely to less likely based on the presented information.

# Pulmonary embolism in COVID-19. When nothing is what it seems

A 61-year-old former male smoker, with a history of hypertension treated with angiotensin-converting enzyme inhibitors, attended the emergency room complaining of dyspnea after a 1-week history of dry cough. His blood pressure was 125/90 mmHg, heart rate 136 bpm, respiratory rate 30 bpm, and oxygen saturation (SatO2) < 85% on room air. Physical examination showed signs of hypoperfusion and use of accessory respiratory muscles. The patient have tachycardia. Eventually, persistent hypotension and desaturation developed, requiring crystalloid solution expansion, as well as noradrenaline and dobutamine.

# Bilateral pulmonary embolism without deep venous thrombosis was observed after knee arthroscopy: a case report

A 50-year-old female patient with no other risk factors other than hypertension, obesity, varicose veins in the ipsilateral lower extremities and elevated triglyceride (TG) presented to the emergency room. The patient had no malignancy or venous thromboembolism (VTE) history; she had no use of anticoagulants or hormones.  Two days ago, knee arthroscopy in the right knee cavity was performed under lumbar anaesthesia. The patient was operated on in a suspended-leg position.  The patient did not receive any pharmacologic thromboembolic prophylaxis. She left the bed and walked on the first postoperative day, and she experienced sudden chest tightness, polypnea and fainting after going to the bathroom the morning of the second postoperative day.  Vitals were peripheral oxygen saturation (SpO_2_): 92%, HR: 98 times/min, R: 20 times/min, Bp: 103/51 mmHg.

# Using Non-Invasive Respiratory Monitoring for COVID-19 Pulmonary Embolism Diagnosis (50).

A 62-year-old man presenting with SOB was admitted to the emergency department (ED) of an urban community hospital. Fifteen days before presentation to the ED, the patient tested positive for COVID-19, and subsequently developed cough, fever, malaise, and diarrhea 4 days after. The patient had a relatively benign medical history that included controlled hypertension, a body mass index of 28, and a remote history of a cholecystectomy. Medications taken at home before admission to the ED included Losartan, vitamins C and D, and melatonin. At the admission to the ER department, the patient was hypoxemic, with an oxygen saturation (SpO2) of 72% on room air, and tachypneic, with a respiratory rate (RR) of 24 breaths/min. The rest of his vital signs were within normal limits, with a blood pressure of 140/84 mmHg, a pulse of 98 bpm, and an oral temperature of 36.7°C.

# Pulmonary embolism as the primary presentation of IgA vasculitis

A 47-year-old man with a medical history of type II diabetes mellitus and hypertension presented to the hospital with sudden onset of shortness of breath. He also mentioned that 1 week ago, he noticed swelling of his right leg and a painful erythematous skin rash on both lower limbs.

A review of systems was positive for mild non-specific diffuse abdominal pain of the same duration. He denied smoking, alcohol drinking or illegal drug use. He mentioned that he drinks 3 L of water daily and is physically active at baseline. On physical examination, he was hypotensive with blood pressure 82/40, heart rate is over 100, tachypneic with a respiratory rate of 35 breaths/min and oxygen saturation 82% on room air. The physical examination showed clear lung sounds. The abdomen was soft, not distended with diffuse tenderness to palpation. Extremities showed reddish to purple, non-blanchable, tender purpuric lesions on both legs and, to a lesser extent, on the right forearm..

1. [**Acute pulmonary embolism mimicking inferior myocardial infarction.**](https://pubmed.ncbi.nlm.nih.gov/24769824/) **(61)**

A 69-year-old male patient was admitted to a chest center with new-onset chest pain and sweating. He had no history of coronary artery disease. He was a smoker - 30 pack-years - and had poorly controlled hypertension. On the first physical examination, his blood pressure was 120/70 mmHg, pulse 80 bpm/ rhythmic, and respiratoryrate 20/min, and normal heart and lung sounds were present.

# High-risk pulmonary embolism assessed by transthoracic echocardiography: A case report. (59)

A 55-year-old, previously healthy woman, complained of dyspnea and pleuritic chest pain for 40 days, along with transitory (10 minutes) episodes of syncope that had occurred 2 days previously. On admission, her body temperature was 36.0°C; pulse wave was 98 beats/min (bpm); respiratory rate was 18 breaths/min; and blood pressure was 98/61 mm Hg. Slight cyanosis on the lips was observed. Bilateral respiratory movements were identical. Breath sounds were diminished and no moist rales were heard. The heart rhythm was regular, with pulmonary second sound > aortic second sound (P2>A2). No edema was found in the lower extremities.

# Acute bilateral pulmonary embolism in a 21-year-old: is May-Thurner syndrome in our differential? (70).

21-year-old African-American man with no significant past medical history presented to the emergency department with severe left leg pain and swelling of 1-month duration and non-productive cough with some exertional shortness of breath for the past couple of days. He has a sedentary lifestyle, a history of blood clot in one of his uncles, active cigarette smoker (one pack a day) and a remote history of intravenous drug abuse. Admission vitals were remarkable for mild tachycardia 111 bpm. Left calf was swollen and tender compared with the right calf.

# Pulmonary Embolism in a Young Immunocompetent Adult Infected with Cytomegalovirus. Are Novel Oral Anticoagulants an Efficient Alternative? (62).

A 25-year-old male presented to the emergency room with sudden onset of chest pain. One month prior to the admission, he had developed persistent fever and cough, and following detailed assessment, diagnosis of CMV infection was established. There was no history of smoking, alcohol intake or other comorbidities. His temperature was 36.9˚C, blood pressure was 125/55, heart rate 125/min, respiratory rate 22/min with oxygen saturation 100% at 2lt of oxygen.

# Development of pulmonary embolism in a nonhospitalized patient with COVID-19 who did not receive venous thromboembolism prophylaxis (64).

A 32-year-old, overweight (weight, 90 kg; body mass index, 28) African American male with a past medical history significant for asthma (not managed with any medications) presented to the ED of an academic medical center with cough, shortness of breath, diffuse chest pain (associated with cough), fevers, chills, myalgia, and diarrhea that had developed over 5 days. The patient’s vital signs were stable aside from a temperature of 38.4°C., The patient returned to the ED 12 days later with complaints of left-sided pleuritic chest pain lasting 1 day. He reported resolution of shortness of breath, fevers, chills, myalgia, and diarrhea and improvement in cough. He denied any recent hospitalizations, prolonged periods of immobility, trauma, smoking, or a history of cancer or prior VTE. He also denied a family history of VTE but reported a history of stroke in his mother. In the ED, vital signs were normal. heart rate was normal.

# Successful pharmacomechanical intervention with ultrasonic-accelerated thrombolytic catheter for massive pulmonary embolism (81).

An 86-year-old male with history of metastatic prostate carcinoma and hypertension was admitted due to 4-day history of left lower extremity swelling, severe pain and redness that had been progressively worse. Associated symptoms included a 3-day history of dyspnea, progressively worse to the point to be worsened upon minimal activities. He denied orthopnea, paroxysmal nocturnal dyspnea, recent surgeries, prolonged immobilization, fever, chills, cough, hemoptysis or chest discomfort. Physical examination revealed the following vital signs: heart rate 87 beats/min, blood pressure of 111/79 mmHg, 24 respirations/min, and oxygen saturation (O2Sat) at 95% on 2 L by nasal cannula. Cardiovascular examination significant for 2/6 systolic murmur in left mid sternal border, no gallops, splits or rubs. He had 2+ pitting edema up to the upper third of the left calf, no cyanosis; and calves nontender to palpation. while waiting the patient developed severe hypoxemia (O2 needs significantly increased to 15 L while in face mask with reservoir in order to keep O2Sats >92%), with sustained hypotension (75/45 mmHg) with cardiogenic shock due to presumed MPE

# Successful application of extracorporeal membrane oxygenation and pulmonary thromboembolectomy in a patient with a life-threatening pulmonary embolism (93).

A 29-year-old gravida 1 para 1 woman arrived to our emergency room because of progressive dyspnea in the morning that began 2 days after a cesarean section. She had no history of diabetes, hypertension, or other chronic diseases. Her antepartum course was uneventful. She underwent a cesarean section because of a nonreassuring fetal heart rate tracing. She remained in bed until the bladder catheter was removed on the 3rd day, at which time she felt dizzy and short of breath. On arrival, her blood pressure was 112/64 mmHg and pulse rate was 126 beats per minute. She became drowsy and lost consciousness 5 minutes later. She was intubated, mechanical ventilation was initiated, and vasopressin and dopamine were administered; however, she became pulseless 20 minutes later, despite cardiac massage.

# Endovascular management of a case of spontaneous retroperitoneal haematoma complicated with deep vein thrombosis and pulmonary embolism (94).

Our patient, a 55-year-old woman, presented to us with a history of pain and swelling of the left lower limb of 7 days’ duration, pain in the lower abdomen and dyspnoea of 3 days’ duration. The pain abdomen was dull, continuous ache in the hypogastric region with no specific aggravating or relieving factors. Dyspnoea was brought about by minimal exertion and was not accompanied by cough or chest pain. There was no history of prolonged immobilisation, leg trauma or any other risk factor for venous thrombosis. She was a postmenopausal woman not on hormonal replacement therapy or any other drugs.On clinical examination, she had tachycardia (130 beats/min), tachypnoea (34/min) and a blood pressure of 104/70 mm Hg. She was hypoxaemic on room air (oxygen saturation was 88%), which improved with oxygen by face mask. Local examination of the lower limb revealed pitting pedal oedema of the left leg extending until the thigh. There was calf tenderness. The distal pulses of both lower limbs were normal and equal. There were no varicose veins, ulcerations or discolouration of the lower limb. Cardiovascular and other system examinations were unremarkable.

1. **Paradoxical embolism with thrombus stuck in a patent foramen ovale: a review of treatment strategies** **(83).**

A 58-year-old male patient presented to the Emergency Department (ED) because of the persistence, for about two weeks, of fever (maximum 38°C), dry cough and arthralgia. The appearance of chest pain elicited by breathing represented the reason for ED referral. At home he had taken aspirin, paracetamol and antibiotics without effect. Patient’s medical history was significant for arterial hypertension and chronic obstructive pulmonary disease. He was an active smoker (20 pack/year). His home therapy consisted of ramipril and carvedilol. At the ED, blood pressure was normal (125/80 mmHg), pulse rate was 120 bpm, oxygen saturation in room air was 94%, temperature was 38.5°C.

# Massive Pulmonary Embolism Following Varicose Vein Surgery That Was Successfully Treated with Thrombolytic Therapy (117).

A 47-year-old woman with a history of right lower limb varicose vein surgery 7 days previously was admitted to the emergency department with squeezing chest pain that was present for 3 days. She had been followed for hypertension and depression, she was a nonsmoker and her family history was nonspecific. A short period following her admission, she had cardiac arrest and after approximately 3-minute re- suscitation, sinus rhythm was obtained. The patient was then intubated and her vital signs were as follows: body tempera- ture of 36.8°C, arterial blood pressure of 70/50 mmHg and heart rate 138/min.

# Use of point-of-care ultrasound in the management of patients presenting with shock: the treatment implications of an early bedside diagnosis of pulmonary embolism (113).

A 70-year-old woman was brought in by ambulance to a district general hospital following an episode of exertional dyspnoea followed by collapse. She had led a remarkably healthy lifestyle and was not on any regular medication. Her relatives reported abrupt unintentional weight loss. On arrival, she had a patent airway and was conversing but was in extreme shock with a sustained systolic blood pressure of 30 mm Hg. She was tachycardic, had a Glasgow Coma Scale of 15, temperature of 36.4°C, Spo2: 89 and a glucose level of 5.9 mmol/L. heart rate was 110 bpm/min.

# Venous thromboembolism and COVID-19: a case report and review of the literature (103).

A 65-year-old Caucasian male patient presented to the emergency room with a 2-day history of dyspnea on exertion. He also noted experiencing a couple of episodes of diarrhea a few days before his dyspnea started, which resolved on its own. The patient’s wife had recently been diagnosed with COVID-19. The patient denied any fever, cough, chest pain, or lower-extremity edema. His past medical history included type 2 diabetes, hypertension, and hyperlipidemia. His past surgical history included a remote history of arthroscopic knee surgery. No personal history of malignancy was noted. No family history of hypercoagulable disease or thromboembolism was present. The patient had never smoked, denied drinking alcohol, and had previously been taking metformin, amlodipine, and simvastatin when at home. His vital signs included blood pressure of 150/98 mmHg, pulse rate of 113 beats/min, respiratory rate of 18/min, oximetry 97% on room air, and a temperature of 36.5 °C (97.8 °F). Pertinent findings on physical examination included clear breath sounds and a regular, rapid heart rhythm on auscultation. There was no lower-extremity edema or calf tenderness.

# The dilemma in the management of haemodynamically stable pulmonary embolism with right heart thrombus (126)

A woman in her 80’s presented to the emergency department with complaints of acute shortness of breath and sudden onset pleuritic chest pain. Her vitals were remarkable for tachycardia and tachypnea; however, her blood pressure was within normal limits. The next afternoon, there was a sudden drop in O_2_ saturation to ~70%, which was improved to ~80% with supplemental oxygenation, and she also had tachycardia at 100 bpm. can you give me ten possible differential diagnoses, ordered from most likely to less likely based on the presented information

# Thrombus risk versus bleeding risk: a clinical conundrum (109).

A 62-year-old man presented to a rural hospital emergency department after being awakened by sudden onset dyspnoea and central pleuritic chest pain radiating to his scapulae. His medical history included hypertension, osteoporosis and stage III chronic kidney disease. His medications included azathioprine, prednisolone, prazocin, perindopril and alendronate.

The patient was an independent retiree who lived alone at home, with a 40-pack-year history of smoking and moderate alcohol intake. Family history included myocardial infarction at age 60 on the paternal side and no significant medical history on the maternal side. The patient appeared mildly distressed and initial observations demonstrated tachycardia (100 bpm) and hypertension (160/90 mm Hg), while afebrile with an oxygen saturation of 99% on room air. The cardiovascular exam revealed a loud P2, a pansystolic murmur and a palpable second heart sound with a displaced apex beat. The respiratory, neurological and abdominal examinations were unremarkable.

# Respiratory distress in a patient with Klinefelter syndrome: a suspicion of COVID-19 hiding severe pulmonary embolism (107).

a 37-year-old man, of Algerian nationality, with clinically gynecomastia and abdominal obesity (Figure 1), diagnosed as having KS (47, XXY) during the infertility workup (testicular biopsy and karyotype (Figure 2). This patient, arriving in Tunisia 36 hours ago, was admitted to the hospital at the time of the COVID-19 pandemic for dyspnea and acute chest pain that developed few hours prior to admission. On arrival at the emergency room, his blood pressure, pulse and respiratory rate were 130/70 mmHg, 120/minute and 26/minute, respectively. His body temperature was 38.2°C and he was clearly conscious. The auscultation showed rapid pulse and no pulmonary rales. The patient was cyanotic with an oxygen saturation of 87% in room air. COVID-19 infection was suspected in the first place.

# An unexpected case of recurrence of pulmonary embolism in a patient recovered from COVID19 in full regimen dose of direct oral anticoagulant drug (116).

51-year-old man with a recent history of COVID-19, pneumonia and right side PE presented to the Emergency Room (ER) with palpitations and chest pain. blood pressure (BP) 90/60 mmHg, heart rate 97 beats/min, respiratory rate 20 breaths/min, temperature 37.1 °C, normal oxygen saturation (SaO2) on room air. On physical examination, he had an irregularly irregular heart rate, normally transmitted vesicular murmur, no findings suggestive for heart failure (HF). Suddenly, he started to complain shortness of breath. He was found to be in respiratory distress, with hypotension (BP 85/55 mmHg), tachycardia (HR 110), and SaO2 85%. Rr: 4 l/min . Oxygen was placed and, due to persistent desaturation, non-rebreather oxygen mask (15 l/min) was started.

# Anticoagulation in the obese patient with COVID-19-associated venous thromboembolism (131).

61-year-old man with a history of morbid obesity, obstructive sleep apnoea and unprovoked pulmonary embolism presented to the emergency department following an unwitnessed collapse at rest. He was independent in self-care at baseline, however his mobility had declined in recent years and he received assistance with instrumental activities of daily living from his wife and daughter. He was a distant ex-smoker who drank minimal alcohol. He used a continuous positive airway pressure device at night for treatment of obstructive sleep apnoea. His only regular medication was dabigatran 110 mg two times per day, with which he reported full compliance. He had attended the emergency twice in the preceding 9 days with increasing shortness of breath and had been diagnosed with COVID-19 infection on the first occasion. At presentation, he was in respiratory distress with a respiratory rate of 32 and peripheral oxygen saturations of 86% of room air. He was haemodynamically stable with a heart rate of 92 and a blood pressure of 135/74 mm Hg.

# Hampton’s hump, Westermark’s sign and Palla’s sign in acute pulmonary thromboembolism: a rare concurrence (151).

A 40-year-old male, non-smoker, presented with cough and right-sided pleuritic chest pain of 10 days and fever of 1-day duration. His previous medical and surgical history was unremarkable. There was no history of significant illness in the family. General physical examination and vital signs were normal; HR is 72 min/dk, spo2: 98 and rr: 16/ min. There was no oedema or tenderness of both lower limbs. There were fine inspiratory crepitations over the right infrascapular and infraaxillary areas.

# COVID-19 pneumonia with hemoptysis: Acute segmental pulmonary emboli associated with novel coronavirus infection (139).

A 42-year-old male without recent travel and no significant personal or familial medical history presented to the emergency department with worsening chest pain, shortness of breath, and hemoptysis. He had been previously diagnosed with mild COVID-19 infection twelve days prior to presentation. He was managed uneventfully at home until the day of presentation when he developed worsening exertional dyspnea, central pleuritic chest pain, and hemoptysis. On presentation he was afebrile and demonstrated a normal heart rate, blood pressure, and oxygen saturation, but he demonstrated a respiratory rate of 30 breaths per minute. His physical exam revealed mild respiratory distress with bibasilar rhonchi but otherwise no other acute findings.

# A 59-Year-Old Woman with Extensive Deep Vein Thrombosis and Pulmonary Thromboembolism 7 Days Following a First Dose of the Pfizer-BioNTech BNT162b2 mRNA COVID-19 Vaccine (153).

A 59-year-old woman presented to the Emergency Department with a 3-day history of sudden-onset left leg pain. There was no history of trauma, immobilization, surgical intervention, local injections, fever, weight loss, miscarriages, or skin rash. Her medical background included type 2 diabetes mellitus, osteoarthritis, and COVID-19 pneumonia in September 2020, which required hospitalization in the general ward for 1 week, with no residual complications. Her regular medications were metformin 1000 mg 2 times daily and ethinylestradiol 30 mcg+levonorgestrel 150 mcg, a combined oral contraceptive pill (OCP), with no interruption of therapy for the past 20 years. She received her first dose of BNT162b2 mRNA COVID-19 (Pfizer-BioNTech) 7 days before her current symptom’s onset and approximately 7 months after her diagnosis of COVID-19 pneumonia. On physical examination she appeared alert and oriented. She is tachypneic. Her vitals were as follows: temperature 37.1oC, blood pressure 161/99 mmHg, heart rate 106 bpm, respiratory rate 24 bpm, and oxygen saturation 98% on ambient air. The left leg was swollen and warm with calf tenderness. Chest auscultation revealed bilateral vesicular breath with no added sounds. she developed

# A rare myxoma-like right atrial thrombus causing syncope: A case report (157).

A 61-year-old ambulant man presented to our emergency department for sudden loss of consciousness that happened and lasted for 5 minutes without convulsion and incontinence during climbing stairs. In addition, recurrent positional dizziness during the last 10 days was also reported by the patient. He had a past medical history of tuberculosis, rheumatic arthritis, and right femoral neck fracture, but no previous unconsciousness experience. Physical examination revealed no remarkable abnormalities except for the rapid heart rate of more than 100 beats per minute. Spo2: 98. RR: 16 temperature: 36.8

# Deep vein thrombosis with pulmonary thromboembolism in a case of severe COVID-19 pneumonia (177).

A 53-year-old man who has type 2 diabetes mellitus, presented to the emergency department with a history of fever and dry cough for 5 days, swelling of the left leg for 2 days, and shortness of breath and chest pain for 1 hour. On examination, his pulse rate was 138 beats/min, with blood pressure of 138/70 mm Hg, respiratory rate of 30 breaths/min, and oxygen saturation was 64% on room air with a temperature of 98.6℉. His left leg was swollen, and tenderness was present over the calf region. A difference of 5 cm was observed between the left and right calf diameters. On auscultation, bilateral basal crepitation was found. The abdominal, neurological and cardiovascular systems were within normal limits.

# Role of hybrid operating room in surgery for the right atrial thrombus, pulmonary thrombi, and ventricular septal rupture after myocardial infarction (181).

A 62-year-old man presented to the emergency room with the chief complaints of chest pain, breathlessness, and pain in the legs for the past 15 days. He had suffered an anterior wall myocardial infarction 15 days ago, and was thrombolysed with streptokinase. There was a history of swelling and pain in the lower limbs 1 year and 3 months ago, which was diagnosed as deep vein thrombosis (DVT). There was no history of hypertension, diabetes mellitus, or smoking. The patient had tachycardia, HR was 110 bpm.

# Case Report on a Patient with Steinert Disease Complicated by COVID-19 (186).

A 63-year-old man presented a transient loss of consciousness and muscle strength with spontaneous recovery while walking in the street. He was a current smoker and had a past medical history of myotonic dystrophy type 1, also known as Steiner disease, and hiatal hernia. The patient denied fever, cough, sputum production, dyspnea, chills, muscle pain, loss of taste/smell or any other symptoms. He also denied excessive decrease in physical activity during the last week or any other risk factor for venous thromboembolism (VTE). Vital signs at admission were: arterial blood pressure 123/93 mm Hg, temperature 35.6℃, heart rate 115/minute, respiratory rate 23/minute, and oxygen saturation 94% at room air. His physical examination revealed a contusion and hematoma in right supraciliary arch, shallow breathing without respiratory distress. The rest of the physical examination was unremarkable.

# Factor XII (Hageman Factor) Deficiency: a rare harbinger of life-threatening complications (210).

## A 29 year old Saudi female with a history of uneventful C/S history 3 three weeks ago presented to the emergency with a sudden severe breathlessness and chest pain. she was in obvious respiratory distress. She has been ambulant and had no past history of being non-smoker, no history of taking any medications. She denied family history of any blood disorders. patient looking anxious and in distress, BP = 105/60 mmgh, HR = 115/min, RR = 29/min, O2 saturation at room air at rest = 85%, Temperature = 36.7C, Weight= 58.7 Kg. No clinical signs of deep vein thrombosis.

# Lessons in clinical reasoning - pitfalls, myths, and pearls: a case of chest pain and shortness of breath (191).

A 57-year-old man was sent to the emergency department from clinic with chest pain, severe shortness of breath, weakness, and “cold sweats”. The day of admission, he was walking up a hill and experienced a non-radiating, dull, substernal chest pain associated with dyspnea, diaphoresis, a “fatigued” feeling, and a “total body weakness”. He went to his truck to rest, which only partially relieved the pain. After several minutes, he tried walking again but the chest pain and dyspnea immediately recurred. This unusual episode worried him so he presented to clinic for evaluation. He recalled intermittent episodes of chest tightness with exertion the previous winter that lasted for seconds and resolved spontaneously. He denied any other prior chest pain or dyspnea, as well as any orthopnea, paroxysmal nocturnal dyspnea, or swelling/pain in his legs. He denied any recent fevers or chills. He had a past medical history of diet/exercise-controlled hypertension and hypercholesterolemia. He took no medications. His past surgical history included bilateral carpal tunnel release. He had no significant family history. He is a never-smoker, drank alcohol socially a few times per year, and denied prior illicit drug use. In the emergency department, he appeared anxious and repeatedly stated “I do not feel well”. His initial blood pressure was 147/90, pulse 102, temperature 36.7, oxygen saturation on room air was 89%, and he was breathing 18 times per minute. On exam, jugular venous pressure was not elevated. Precordial auscultation revealed tachycardia with a regular rhythm, a normal S1 and S2, and the absence of murmurs, rubs or gallops. Radial and dorsalis pedis pulses were 2+ bilaterally. Lung exam demonstrated slightly increased work of breathing without accessory muscle use, but was otherwise normal. His abdominal examination was benign. The lower extremities lacked edema. There were no neurologic deficits. The patient became dyspneic after walking only a few steps and had to sit down. A 12-lead ECG showed sinus tachycardia with T wave flattening in leads V2–V4 and occasional premature ventricular contractions. he was diagnosed with NSTMI and received coronary angiography. The day after discharge, the patient developed recurrent chest pain, shortness of breath, and diaphoresis as he was walking around his house. He again presented to an outside facility for these symptoms and was noted on arrival to have a new 4-L oxygen requirement to keep his oxygen saturations above 90%.

# Recurrent pulmonary embolus despite adequate anticoagulation: the case for routine cancer screening, prompted by an uncommon cause (237).

A patient in their 65s male presented to a hospital with a week-long history of worsening dyspnoea and a marked reduction in exercise tolerance to 30 yards. There was concomitant fresh frank haemoptysis, fatigue, anorexia and unintentional weight loss of 9 kg over the course of a month. This was in the context of a previous admission a month beforehand, where dyspnoea and hypoxia was shown to be due to a PE on CT pulmonary angiogram. The patient was treated with warfarin, with a target INR set between 2 and 3. This was within the target range on readmission. Other history included chronic lymphocytic leukaemia (CLL) stage 0, mumps and tonsillectomy. The patient’s mother was diagnosed with breast cancer in her 60 s. On examination, there were reduced breath sounds in the right upper zone of the lung. The jugular venous pulse was elevated at 6 cm from the sternal angle. The patient had a tachycardia of 105 bpm, a blood pressure of 70/50 mm Hg, a respiratory rate of 30 breaths per minute and saturations of 94% on room air.

# A 75-year-old with abdominal pain, hypoxia, and weak pulses in the left leg (243).

A 75-year-old man presented to the emergency department for evaluation of abdominal pain. He had stage 3 chronic obstructive pulmonary disease. Aside from his COPD, he had been healthy until 1 month earlier, when he had been hospitalized because of shortness of breath and chest pressure with exertion. Left heart catheterization had shown no significant coronary artery disease. in the emergency, the patient described persistent and severe periumbilical abdominal pain during the previous day. It was not associated with eating, and he denied diarrhea, constipation, hematemesis, hematochezia, bright red blood per rectum, or melena. He continued to describe persistent shortness of breath and pleuritic chest pain. His vital signs were as follows: Heart rate 104 beats per minute, Respiratory rate 16 to 20 breaths per minute, Blood pressure 101–142/62–84 mm Hg, Oxygen saturation 78% on room air. On examination, his lungs were clear bilaterally. His abdomen was diffusely tender but without peritoneal signs. His left lower leg was cool to touch, and his left dorsalis pedal and posterior tibial pulses were only weakly palpable. His right leg pulses were normal. He denied pain in the lower extremities. No jugular venous distention was noted, and cardiac examination was most notable for tachycardia. HR: 110 BPM.

# Tracheal stenting on venovenous ECMO with a dual lumen right atrium-to-pulmonary artery cannula in a COVID-19 patient with an inferior vena cava filter (244).

# At the beginning of March 2021, a 47-year-old man, with no medical history, tested positive for Sars-CoV-2. After 7 days, he was brought to the emergency department with cough and shortness of breath. At admission, peripheral oxygen saturation was 45% and increased to 85% with oxygen mask, respiratory rate was 45 breaths per minute, arterial pressure 116/72 mmHg, and heart rate 127 beats per minute. History of thromboembolism, hemoptysis, recent surgery and leg pain/swelling was not present

# Pulmonary embolism, transient ischaemic attack and thrombocytopenia after the Johnson & Johnson COVID-19 vaccine

Our patient is a 43-year-old Caucasian woman with a history of hyperlipidaemia, anxiety, depression, obesity, obstructive sleep apnoea and gastro-oesophageal reflux disease who presented to the emergency department with a 3-day history of generalised headache, fever, body aches, chills, mild dyspnoea and lightheadedness. Approximately 10 days before her presentation, she received the J&J COVID-19 vaccine. She reported a maximum temperature of 103.8°F. She decided to seek evaluation due to the progressive nature of her headache, which had become unbearable to the patient at the time of her evaluation. ital signs were stable with a blood pressure of 124/80 mm Hg, heart rate of 65 beats/min, a temperature of 37.9^o^C, a respiratory rate of 20 breaths/min and an oxygen saturation of 96% on room air. On respiratory examination, the patient had good air entry bilaterally with no wheezing throughout the lung fields and no crackles at the lung bases. Neurological examination was within normal limits, with no focal motor or sensory deficits.

# COVID-19 and Acute Pulmonary Embolism: A Case Series and Brief Review

A 38-year-old man with no significant past medical history and a recent outpatient diagnosis of COVID-19 infection was brought to the emergency department after a syncopal episode at work. He had tested positive for COVID-19 infection by polymerase chain reaction test two weeks prior to presentation. Over the week prior to presentation, he developed a gradually worsening cough, shortness of breath, decreased appetite, fatigue, and diffuse muscle aches.

At presentation, he appeared ill, restless and diaphoretic with an increased work of breathing. His respiratory rate was 40/min, oxygen saturation 95% on a100% non-rebreather, and he was hypotensive with a systolic blood pressure of 78 mmHg. HR: 112 bpm.

# A unique tale of COVID-19 induced concomitant overt disseminated intravascular coagulation and acute bilateral pulmonary embolism

A priority call was initiated in the emergency triaging area when paramedics brought in a 65-year-old woman with an oxygen saturation of 65% on room air. She was given full flow oxygen at 15 L through a non-rebreathing mask, which elevated her saturation to 92–95%. Upon stabilising the patient, history revealed that she had been experiencing viral prodrome for the past 7 days. Her symptoms were primarily fever, chest pain, cough with clear expectoration, and intermittent shortness of breath on exertion. She had been monitoring her oxygen saturations at home and noted them to be approximately 60–65% on room air. She had no gastrointestinal symptoms nor smell/taste alterations that are common with Severe Acute Respiratory Syndrome Coronavirus 2 (SARS-COV2). Her past medical history included Type II Diabetes Mellitus and she was taking oral hypoglycemics. She reported being otherwise fit and well and a non-smoker.

During a general examination, the patient was noted to have pleuritic sounding chest pain and was markedly dyspnoeic, tachycardic and tachypnoeic, and had an oxygen saturation of 92–95% on a 15 L non-rebreathing mask. She was unable to speak in full sentences due to breathlessness. Auscultation during chest examination revealed pleural rub along with bi-basal crackles without any wheeze. Other systemic examinations were normal.

# When I treat a patient with acute pulmonary embolism at home

# A 58-year-old woman was evaluated in our hospital because of acute dyspnea and pleuritic chest pain. Symptoms had started 1 week before presentation. She reported no provoking factors for PE nor symptoms suggestive of deep vein thrombosis. Her temperature was 37.2°C, heart rate was 85 beats/min, respiratory rate was 14 breaths/min, oxygen saturation at room air was 98%, and blood pressure was 136/72 mm Hg. Her physical examination were unremarkable.

# An uncommon presentation of COVID-19: concomitant acute pulmonary embolism, spontaneous tension pneumothorax, pneumomediastinum and subcutaneous emphysema (a case report)

A 55-year-old man with a previous history of erythroblastopenia due to thymoma having undergone thymectomy followed by radio-chemotherapy and long-term corticosteroid therapy presented to the emergency department (ED) of our center after 15 days of fever, dry cough, asthenia, myalgia, headache with marked dyspnea. The patient was hemodynamically stable (blood pressure at 140/70mmhg, a heart rate at 80 beats/min) with an increased breathing (tachypnea 25 breaths/minute, oxygen saturation at 70% in room air), and a high temperature (38.2°C). The physical examination revealed subcutaneous emphysema in the chest and neck.

The patient tested positive for SARS-CoV-2 reverses transcriptase-polymerase chain reaction test (RT-PCR) performed on nasopharyngeal swabs. high D-Dimer level

# Stroke and refractory hypoxaemia: complications of pulmonary embolism

A 46-year-old woman was admitted to the emergency department with sudden aphasia that started less than an hour prior. She had been submitted to bursectomy of the left knee 2 weeks before, with no heparin prophylaxis afterwards, and had no other relevant medical history besides taking oral contraceptives.

On initial examination, the patient was alert and followed simple commands but was unable to answer simple questions. She presented no other neurological findings (initial score on the National Institutes of Health Stroke Scale (NIHSS) 4/42 for expressive aphasia). She presented tachycardia heart rate 118 beats/min, arterial blood pressure 116/71 mm Hg, respiratory rate of 22 breaths/min, oxygen saturation of 71% on air and was afebrile.

# Controversy and consent: achieving patient autonomy in thrombolysis for acute submassive pulmonary embolism

A 59-year-old woman presented on the acute medical take with worsening shortness of breath over a period of 3 weeks. She described a history of shortness of breath initially on exertion; however, this became increasingly severe and, at the time of presentation, even occurred at rest. She described right-sided pleuritic chest pain coinciding with her dyspnoea, but in the absence of syncope, palpitations or central chest pain. There was neither haemoptysis, nor calf swelling, previous DVT/PE, family history of thrombosis/recent travel, nor was there immobility. The patient had no ‘red flag’ symptoms suggestive of underlying malignancy. Her medical history included hypertension, osteoarthritis and primary hyperthyroidism. She took losartan 100 mg once daily and levothyroxine 75 μg once daily. There were no reported drug allergies. She was independent and lived with her partner.

On examination, she was dyspnoeic at rest, with a regular heart rate of 95 bpm. She had an oxygen requirement (2 L via nasal specs) to maintain SpO_2_ between 94% and 98%. Her heart sounds were normal and jugular venous pressure was not visibly raised; she had a blood pressure of 148/91 mm Hg. No other abnormalities were found on examination other than a large body habitus.

# The Dilemma of the “Ischemic-looking” Electrocardiogram: Pulmonary Embolism or Acute Coronary Syndrome?

# The patient was a 45-year-old obese female who presented with sudden onset chest pain and shortness of breath for 2 days. She was a nonsmoker and had no previous history of hypertension, diabetes, or coronary artery disease or use of any medications; a 12 lead ECG done outside revealed T-wave inversion in anterior precordial leads and elevated Troponin T levels (0.33 ng/ml, upper limit of normal 0.01 ng/ml). On presentation at our institution, she was dyspneic, with a respiratory rate 26/min and blood pressure of 110/70 mmHg. Chest and cardiovascular examination was unremarkable, without any evidence of heart failure or pulmonary congestion.

# Extensive DVT and Pulmonary Embolism Leading to the Diagnosis of Coronavirus Disease 2019 in the Absence of Severe Acute Respiratory Syndrome Coronavirus 2 Pneumonia

# A 31-year-old female patient with no past medical history presented to the ED for an erythematous swelling of the left inferior limb. The patient had no fever, stable hemodynamic status, and oxygen saturation was 99% (room air). Notably, the heart rate was 133 beats/min. There was no sign of coronavirus 2019 disease (COVID-19) pneumonia, nor evidence of solid or hematologic malignancy. The patient had no risk factors and no familial history of VTE. However, her tachycardia is a big concern.

# Acute massive pulmonary embolism: role of the cardiac surgeon

A 72-year-old woman underwent right hemicolectomy and pancreaticoduodenectomy for locally advanced colonic adenocarcinoma. She became acutely cyanotic and went into acute respiratory distress 6 hours postoperatively. She was also hypotensive (systolic blood pressure as low as 40 mmHg) and cyanotic (SaO_2_ of 50% and PaO_2_ of 46.8 mmHg), hr: 111 and had to be intubated and started on high-dose inotropic support.

blood pressure of 63/39 mmHg and a central venous pressure of 28 mmHg on neosynephrine, epinephrine, dobutamine, and vasopressin support. The patient did not require cardiac massage throughout the resuscitative efforts.

# Correlation Between ST‐Segment Elevation and Negative T Waves in the Precordial Leads in Acute Pulmonary Embolism: Insights into Serial Electrocardiogram Changes

# A 63‐year‐old female was admitted to Shiyan Taihe hospital due to progressive dyspnea, tightness in the middle chest and repetitive syncope for 5 days. She had no history of cardiopulmonary disease. The blood pressure (BP) was 80/50 mmHg. She didn’t report hemoptysis, history of recent surgery/immobilization and leg swelling/pain.

# Tricuspid valve thrombus causing acute pulmonary embolism

A 74-year-old woman was admitted to our hospital with the signs of dyspnea, chest pain, palpitations, and dizziness. She gave a history of pulmonary embolism and deep venous thrombosis (DVT) 5 years ago. Her physical examination revealed a blood pressure of 110/80 mm Hg and a heart rate of 82 beat/min. She had a mild systolic murmur heard best at the lower left sternal border. The patient was inintiallt haemodynamically stable. However, on the second day of her hospitalisation, the patient showed progressive clinical deterioration. She became tachypneic and tachycardic with low blood pressure. ta:80/60 nb:112 spo2:96 rr: 22

# Electrocardiographic findings in pulmonary embolism

# A 57-year-old woman with a history of hypertension and a recent haemorrhagic stroke was admitted with an acute onset of shortness of breath for a duration of one day. She also reported an episode of near syncope while engaging in physiotherapy. At the emergency department, her initial vital parameters included blood pressure of 139/97 mmHg, heart rate of 99 beats per minute (bpm) and oxygen saturation of 89% on room air.

# Acute adrenal failure following anticoagulation with dabigatran after hip replacement and thrombolysis for massive pulmonary embolism

A 75 -year-old woman presented to the emergency department with sudden onset of shortness of breath. This was associated with fever, and had been worsening over several hours. She had been discharged from the hospital, earlier that day, 9 days post-total hip replacement and had been discharged with prophylactic dabigatran 150 mg one time a day.

The patient had a medical history of obliterative bronchiolitis, treated with inhalers and was on no other regular medications. She lived independently.

On examination, she had a fever of 39.4 centigrade and was tachycardic. Her blood pressure was 120/57. Respiratory rate was 30 and oxygen saturations were 93% on 2 litres of oxygen.

# Cytomegalovirus infection with pulmonary embolism, splenic vein thrombosis and monoclonal gammopathy of undetermined significance: a case and systematic review

# A 62-year-old woman with an unremarkable medical history arrived at the emergency department with dry cough and sternal pain that worsened during inspiration. She had already been suffering from headache, nausea, nocturnal sweating and fever of up to 39.4°C for 16 days before presentation. There had been no response to antibiotic treatment with azithromycin and doxycycline prescribed by her general practitioner. The patient did not smoke, drank one or two glasses of wine daily and did not use recreational drugs. There were no family members with similar symptoms. On physical examination, the patient was alert and fully oriented. She was feverous with a temperature of 38.6°C. Her blood pressure was 131/73 mm Hg, with a pulse of 108 beats per minute. The peripheral oxygen saturation was 96%, with a respiratory rate of 16 breaths per minute while breathing ambient air. During auscultation of the lungs, a pleural friction rub was heard in the left lower region with otherwise normal inhalation and exhalation sounds. On further physical examination no additional abnormalities were found.

# Life-Saving Systemic Thrombolysis in a Patient with Massive Pulmonary Embolism and a Recent Hemorrhagic Cerebrovascular Accident

a 60-year-old woman who had sustained a hemorrhagic cerebrovascular accident (CVA) 8 weeks earlier presented with acute lethargy, blurred vision, hypotension, and bradycardia.Further evaluation yielded deep vein thrombosis in the right lower extremity. On the 3rd day of hospitalization, the patient had 2 episodes of cardiac arrest with pulseless electrical activity.

# A Rare Case of Submassive Pulmonary Embolism with a Right Aberrant Subclavian Artery and Thrombosed Kommerell Diverticulum

An 83-year-old man presented at a nearby hospital with shortness of breath. He had a history of hypertension, dyslipidemia, and hyperuricemia but no history of embolism. He was diagnosed with bronchial asthma, and treatment was initiated with oral medication and an inhaler. However, his symptoms worsened, and at a subsequent consultation he had hypoxemia, with an oxygen saturation (SpO_2_) of 92% in room air. On admission, the patient's heart rate was 94 beats per minute, and his blood pressure was 131/74 mmHg. His hemodynamics were stable. His heart and lung sounds were normal, and no leg swelling was detected.

# Pulmonary infarction in acute pulmonary embolism: reversed halo sign

A 24-year-old female patient was admitted with symptoms of right lower limb swelling, shortness of breath and dry cough of 1 week duration. There was no history of fever, chest pain, oral contraceptive use, smoking or underlying malignancy. On examination, heart rate was 100 beats per minute, respiratory rate was 24 breaths per minute while other vital signs and systemic examinations were normal except for swelling in the right lower limb. Oxygen saturation on room air was 94%.

# Massive Pulmonary Embolism in a Patient with Heparin Induced Thrombocytopenia: Successful Treatment with Dabigatran

A 57 years-old female patient was referred to our clinic with the diagnosis of mPTE. She had shortness of breath and chest tightness and suffered from syncope attacks. She was obese, hypertensive for 14 years and received LMWH (enoxaparin 0.4, 1×1 subcutaneously) due to prophylaxis of unilateral total knee replacement for 11 days.

On the admission day to our clinic, her physical examination showed that TA: 90/60 mmHg, pulse: 102/min., fever: 36.4°C, respiratory rate: 44/min., O_2_Sat: 88% and, a systolic murmur at tricuspid valve. All other physical system examinations were within normal limits.

# A 25-Year-Old Woman with a High-Risk Large and Occlusive Pulmonary Embolism, Later Diagnosed with Primary Antiphospholipid Syndrome and Hyperhomocysteinemia: A Case Report (37)

A 25-year-old woman was admitted to the Emergency Department with sudden-onset dyspnea after elective cholecystectomy. One year earlier, she was diagnosed with lower extremity deep venous thrombosis (DVT) without an identified predisposing cause and was treated with a vitamin K antagonist (VKA) for 6 months. She had no history of hypertension, diabetes, hypercholesterolemia, obesity, or use of combined oral contraceptives. There was no family history of hemato-logic or autoimmune conditions. Her initial blood pressure was 95/60 mmHg, pulse rate was 94 beats/min, respiratory rate was 24 breaths/min, and oxygen saturation was 95% on oxygen 3 L/min. On physical examination, she had right lower extremity edema.

# Pulmonary Embolism Associated with Clomiphene Citrate in a Young Woman

A 28 year old woman was admitted to the emergency department of our hospital for chest pain ongoing for one day. There was no feature of Pulmonary Embolism, in the patient’s medical history. Treatment of ovulatory dysfunction with clomiphene citrate (50 mg once a day) had been initiated 20 days prior. In her physical examination, blood pressure was 100/60 mmHg, heart rate was 120 bpm. Other systemic and laboratory findings were normal.

# A rare case of severe pulmonary embolism revealed by consciousness disorders

A 33-year-old woman presented with acute breathing difficulties, purple lips and sweating. She had no cough, hemoptysis and chest pain. The patient was unconscious when the ambulance arrived. The physical examination revealed that she had a blood pressure 80/50 mmHg, blood oxygen saturation 88% on air. HR: 120, RR: 35 SPO2: 88. The patient suddenly appeared full body convulsions, decreasing heart rate and then cardiac arrest.

1. **A 44-Year-Old Woman With Chest Pain and Dyspnea**

A 44-year-old woman with a medical history of anti-phospholipid antibody syndrome complicated by recurrent pulmonary emboli with subsequent chronic hypoxic respiratory failure (3 L/min oxygen baseline) presented to the ED with 2 to 3 weeks of shortness of breath and pleuritic chest pain that radiated to the center of her back. These symptoms were accompanied by an increase in her oxygen requirement from 3 L/min to 6 L/ min. She also reported nausea, vomiting, lightheadedness, and dizziness for the same period. The patient had two prior pulmonary emboli in the same year, which prompted a hypercoagulable workup, ultimately revealing a diagnosis of antiphospholipid antibody syndrome. The second pulmonary embolus occurred while the patient was on coumadin, though achieving a therapeutic international normalized ratio was challenging. At the recommendation of the Hematology Department, she was transitioned to systemic anticoagulation with low-molecular- weight heparin (LMWH) at a dose of 1.5 mg/kg twice daily, which was her regimen at the time of admission. The patient confirmed total compliance with her anticoagulation therapy, and she denied any recent travel or long periods of being sedentary. She was up to date on her age- appropriate cancer screening, without any evidence of active malignancy.

Vital signs at the time of admission included a temperature of 36.2 C, a heart rate of 86 beats/min, BP of 130/81 mm Hg, an oxygen saturation 96% on 6 L/min nasal cannula, and a respiratory rate of 16 breaths/min. The patient was mildly distressed, with subtle accessory muscle usage with breathing. She had jugular venous distension to the level of her earlobe with the head of the bed at 45 degrees. She had a normal rate and regular rhythm without significant murmur. Her lungs were clear to auscultation with limited expansion of the chest due to pain. Her abdomen was soft and nontender. She had 2þ lower extremity pitting edema bilaterally.

# Massive hemoptysis in a patient with pulmonary embolism, a real therapeutic conundrum

A 45-year-old non-smoker male patient presented to the [emergency department](https://www.sciencedirect.com/topics/medicine-and-dentistry/emergency-department) of a territorial hospital complaining for [hemoptysis](https://www.sciencedirect.com/topics/medicine-and-dentistry/hemoptysis). On the day of admission, he reported that he had expectorated approximately 50ml of bright red blood. He denied any other symptoms, including fever, cough, dyspnea or [chest pain](https://www.sciencedirect.com/topics/medicine-and-dentistry/thorax-pain). On his [medical history](https://www.sciencedirect.com/topics/medicine-and-dentistry/medical-history) he reported a similar episode of hemoptysis 5 years ago the cause of which remained unknown despite clinical investigation which included [computed tomography](https://www.sciencedirect.com/topics/medicine-and-dentistry/computer-assisted-tomography) (CT) of the chest, immunologic examinations and [fiberoptic bronchoscopy](https://www.sciencedirect.com/topics/medicine-and-dentistry/fiberoptic-bronchoscopy). The patient denied any other [medical problem](https://www.sciencedirect.com/topics/medicine-and-dentistry/disease) and was not receiving any medication.

On physical examination, he was a pleasant apparently healthy man with body temperature 36,8 °C, pulse rate 110 beats/min, blood pressure 130/80 mmHg, respiratory rate 21 breaths/min and [oxygen saturation](https://www.sciencedirect.com/topics/medicine-and-dentistry/oxygen-saturation) 95% on room air. [Auscultation](https://www.sciencedirect.com/topics/medicine-and-dentistry/auscultation) disclosed mild crackles in the right lower lobe. No other abnormal findings were found in the rest of the physical examination.  .

# Atypical Presentation of Pulmonary Embolism Several Months After COVID-19 Infection

A 47-year-old morbidly obese, non-smoker African American female with a past medical history of hypertension and COVID-19 infection (in April 2020), presented to the emergency in late November after having a sudden episode of dizziness while at work. The patient reported that she went to work at around 11 pm and was fine till 1:30 am when she abruptly developed dizziness associated with severe nausea and three episodes of non-bilious vomiting. She described the episode as the sensation of everything in the room spinning around her. The review of the system was otherwise unremarkable. She denied a history of prolonged immobilization, recent surgery, personal or family history of blood clots, and was not taking oral contraceptives or hormonal supplements. She was a non-smoker. She denied chest pain, shortness of breath, palpitations, cough, change in vision, headache, pain in the extremities, and syncope. Of note, after testing positive for COVID-19 six months before presentation, the patient improved with supportive treatment without the need for hospitalization. She was diagnosed with systemic hypertension three years ago for which she was taking valsartan and furosemide. She had a history of knee replacement surgery in 2012 without any post-operative complications. She was a nurse by profession. There was no family history of blood clots. On physical examination, her blood pressure was 186/121 and her oxygen saturation was 98%-99% on room air. HR: 69 BPM.

# Acute pulmonary embolism with loss of consciousness as the first manifestation: a case report

The patient was 50-year-old male, who 2 days before admission, had difficulty breathing when standing at home after bending over and working, which was accompanied by shortness of breath and amaurosis. He subsequently fell to the ground unconsciousness but experience no fall injuries. After being unconscious on the ground for 10 seconds, the patient regained consciousness and had experienced no convulsion when falling, no rolling up of the eyes, and no incontinence. There was no dizziness, headache, chest tightness, chest pain, nausea or vomiting, palpitation, or sweating before the onset of loss of consciousness. After waking up, the patient continued to have dyspnea, general fatigue, intermittent shortness of breath, and chest pain. After that, the patient developed intermittent dry cough but no fever or expectoration, no dizziness or headache, no palpitation or back pain, and no residual neurological symptoms and was able to walk. He complained that when he went up the stage, he had increased shortness of breath and amaurosis but no chest pain or fainting. Moreover, the patient had no history of chest distress, chest pain, or asthma. He could lie down quietly and sleep at night. For further diagnosis and treatment, he visited to the cardiology department of Beijing Anzhen Hospital. From the onset of the disease, the patient had a clear mind, good spirits, normal stool and urine, and no significant change in weight. He had a history of gout for about 3 years. Before admission, he had experienced gout attacks and swollen and painful feet. The patient had oral administration of Celebrex and sodium bicarbonate 1 month before admission which reduced the activity of both his lower limbs. He had a history of varicose veins of the lower limbs for 20 years and a history of fatty liver for 10 years. The patient underwent radiofrequency ablation for paroxysmal atrial fibrillation 3 years prior in our hospital, and there was no recurrence during postoperative follow-up. His father had a history of high blood pressure, diabetes, and pulmonary embolism, while his mother had coronary heart disease. Physical examination results on admission were the following: temperature, 36.5 ℃; pulse, 82 times/minute; respiration, 16 times/minute; and blood pressure, 131/74 mmHg. The superficial lymph nodes of the whole body were not palpable or swollen. The breath sounds in both lungs were clear, no dry or wet rales were heard, the heart boundary was normal, the heart rhythm was regular, and no pathological murmur could be heard in the auscultation area of each valve. P2 showed no hyperactivity, the abdomen was flat and soft with no tenderness, there was no rebound pain or muscle tension, and bowel sounds were normal. The patient had mild edema in both the lower limbs but was negative for gastrocnemius gripping pain. Other physical examinations showed no obvious abnormalities.

# A 66-year-old woman with high-risk pulmonary embolism

Emergency medical services (EMS) were called to the house of a 66-year-old female patient for respiratory distress. Per the patient’s family, her past medical history was significant for hypertension, anemia, and sleep apnea. She was diagnosed with primary lung adenocarcinoma and subsequently under- went a left upper lobectomy in 2013. Additionally, she had been diagnosed with deep vein thrombosis and PE in the remote past. It is unclear whether the history of these proco- agulative diagnoses was secondary to her history of lung can- cer. She had been prescribed and was currently taking warfarin. The patient had difficulty breathing the morning of the call and was found diaphoretic in the restroom where the family found her after hearing a fall. The patient had been recently evaluated for lower extremity swelling. An ultra- sound was performed that did not demonstrate any evidence of a venous thrombus. At the time of evaluation, she was noted to have an international normalized ratio of 1.2 (thera- peutic range for PE is 2.0-3.0).

On EMS arrival, the patient was in marked respiratory dis- tress and was placed on a nonrebreather mask, but accurate oxygen saturation was unable to be obtained. The patient was alert and oriented. During transport, the patient became more tachypneic and less responsive. Subsequently, she was transi- tioned to continuous positive airway pressure for additional respiratory support. Oxygen saturation could never be obtained during transport, but she was noted to be tachy- cardic in the 100s and hypotensive. When EMS arrived in the resuscitation bay of the local com- munity hospital, the patient was found to no longer be respon- sive and was in pulseless electrical activity arrest. Chest compressions were initiated, and the patient was intubated with initial saturation of 60%. eck. Bedside ultrasonography after ROSC showed a dilated right ventricle, and the prehospital electrocardiogram (Fig. 1) indicated right ventricular strain.

1. **THROMBOLYSIS IN ELDERLY PATIENTS WITHMASSIVE PULMONARY EMBOLISM**

# An 82-year-old woman presented with syncope after a12-hour-long train journey. Ten years before, she had beentreated with radioiodine for multinodular goiter, and 5years before she had had a transient ischemic attack. Shealso had osteoporosis. She was receiving alendronic acid. vitamin D, aspirin, and omeprazol. Her functional andcognitive status was normal (Barthel Index 95/100; PfeifferIndex 1 mistake out of 10). In the emergency department,her blood pressure was 60/40 mmHg, breath rate 30 perminute, and heart rate 110 beats per minute.

1. **Invasive pulmonary aspergillosis/pseudomonas 1**

A 47-year-old Caucasian man presented to the outpatient pulmonary clinic with dyspnoea and chest discomfort for 3 days. He denied cough or haemoptysis. He had a 20 pack-year history of cigarette smoking, chronic obstructive pulmonary disease and an area of chronic necrotising, cavitary aspergillosis (previously diagnosed by bronchoalveolar lavage culture) with an aspergilloma. He had been on chronic daily antifungal treatment with voriconazole for 6 years. Physical examination revealed a thin man in no distress with bilateral equal breath sounds. **No tachycardia**

Diagnosis: angioinvasion into the left main pulmonary artery

# Feather duvet lung 2

In November 2016, a 43-year-old man presented to his general practitioner (GP) with a 3-month history of malaise, fatigue and breathlessness. These symptoms were initially attributed to a lower respiratory tract infection. He had no other respiratory symptoms. His symptoms improved initially, but later that month recurred, such that he had to take 14 days off work. He was a non-smoker, and normally well, apart from occasional sciatica and tinnitus. His GP arranged initial blood tests which were all normal (full blood count, renal function, liver function and C reactive protein). In December 2016, he described worsening breathlessness.  By now he was describing breathlessness walking between rooms at home. His GP referred him for an urgent respiratory clinic review and also phoned for advice. During the phone discussion, one of the authors (OJD) reviewed the patient’s CXR and disagreed with the original report. OJD phoned the patient for more clinical information and noted that the patient sounded alarmingly tachypnoeic on answering the phone. His history was reviewed and the patient described living in a warm dry house with his wife. They had a loft which he rarely entered. They had an en-suite bathroom, and despite an extractor fan, he described a small amount of mould above the shower and window. They had a cat and a dog, but no birds. They had recently acquired a feather duvet and feather pillows, having formerly had synthetic bedding. His hobbies included playing music as part of a band (guitar, saxophone) and his work was office-based with no obvious occupational exposures. His symptoms did not improve when on holiday. He had no relevant foreign travel history or drug history, apart from occasional paracetamol use. **No tachycardia**

diagnosis: hypersensitivity pneumonitis

# Giant pulmonary aneurysm 3

A 47-year-old man was referred to our hospital with progressive irritant dry coughing and dyspnea for 3 weeks. In the previous 4 months, he had experienced that his overall condition and training capacity in sports had decreased considerably. A physical examination revealed chest area systolic jet noise. Laboratory tests showed a normal autoantibody spectrum and PO_2_ of 94.7 mm Hg (normal value 107–116 mm Hg). **No tachycardia**

Diagnosis: pulmonary aneurysm

1. **A 60-Year-Old Woman with a 6-Week History of Shortness of Breath and Intermittent Chest Pain Due to Chronic Thromboembolic Pulmonary Disease Undetected by Computed Tomography Pulmonary Angiography (CTPA) and Diagnosed by Ventilation-Perfusion Imaging (4)**

A 60-year-old woman was admitted to the hospital with a 6-week history of worsening shortness of breath. This was accompanied by a chronic dry cough, and intermittent, central chest pain that was non-radiating and dull in character. The onset of the chest pain was insidious. Importantly, the patient had no history of prior chest pain nor exertional chest pain. As a result of breathlessness at rest, her mobility and exercise tolerance had become greatly reduced, to the point at which she had become housebound. Her past medical history included chronic obstructive pulmonary disease (COPD), for which she had not previously required home oxygen, as well as pulmonary sarcoidosis, obesity, and a 20 pack-year history of smoking. Importantly, she had received all 3 COVID-19 vaccinations prior to admission, and had tested negative for COVID-19 by both rapid antigen testing and by polymerase chain reaction (PCR).

The patient had been assessed by her general practitioner 1 week prior to hospital admission and diagnosed with a presumed chest infection. However, after completing a 7-day course of clarithromycin there was no symptomatic improvement.

Clinical assessment in the ER found the patient to be tachycardiac, with a heart rate of 125 beats per minute (bpm) and with a blood pressure of 135/68 mmHg. At rest she required 3 liters of oxygen to maintain target oxygen saturations of 88-92%, yet would rapidly desaturate to 78% on minimal exertion despite supplemental oxygen therapy. Clear breath sounds were audible throughout both lungs.

Tani: CTEPD

# Hepatocellular carcinoma with thoracic metastases presenting as hemothorax 7

A 60-year-old woman with progression of HCC and lung metastasis was admitted to our hospital for her 12th cycle of chemotherapy with a folinic acid, fluorouracil, and oxaliplatin (FOLFOX) regimen on March 2, 2016. She had a history of HCC in segments 7 and 8 of the liver and received segmental hepatectomy in September 2009; tumor recurrence in segments 5 and 6 with tumor rupture and hemoperitoneum postemergent embolization and segmental hepatectomy in March 2011; local recurrence in the liver dome and lung metastasis after receiving sorafenib from March 2012 to February 2013; She also had diabetes mellitus, chronic hepatitis C, liver cirrhosis, and hypertension.On admission, physical examination revealed a body temperature of 36.6°C, a pulse rate of 106 beats/min, blood pressure of 118/80 mm Hg, and a respiration rate of 18 breaths/min. Two days after admission, she complained of dyspnea

Tani: hemotorax

# A Rapid Change in Pressure 8

A 74-year-old woman presented with a 6-week history of progressive dyspnea on exertion. Eight weeks before this presentation, she had been traveling in Italy and had been walking up to 4 miles per day. Progressive dyspnea on exertion had developed after she returned to the United States. She also noted a nonproductive cough, fatigue, loss of appetite, and an unintentional weight loss of 9 kg over the previous 6 months. She had no fevers, chills, night sweats, hemoptysis, wheezing, chest pain, palpitations, orthopnea, paroxysmal nocturnal dyspnea, swelling of the legs or feet, abdominal pain, nausea, vomiting, melena, or hematochezia. Four weeks before this presentation, she had presented to an emergency department at another institution, where imaging was performed. Chest radiography showed no pneumonia, and venous ultrasonography of both legs showed no deep venous thrombosis. A 5-day course of prednisone and azithromycin was prescribed; however, her symptoms continued to worsen, and she began to have dyspnea when she was at rest. The patient had a medical history of cough-variant asthma (reactive airway disease that manifests as a nonproductive cough), hypertension, and hyperlipidemia. Her medications included albuterol administered through an inhaler, amlodipine, budesonide–formoterol administered through an inhaler, and lovastatin. She had a smoking history of 15 pack-years but had quit smoking entirely 40 years earlier. Her family history was notable for colon cancer in her father and pulmonary hypertension of unclear cause in her mother at the age of 70. There was no family history of autoimmune disorders. On physical examination, her temperature was 37°C, blood pressure 113/69 mm Hg, pulse 108 beats per minute, respiratory rate 24 breaths per minute, oxygen saturation 88% while she was breathing ambient air, and body-mass index (the weight in kilograms divided by the square of the height in meters) 23.5. The mucous membranes were moist, and the nasopharynx and oropharynx had no ulcerations or exudates. Cardiac examination revealed tachycardia, prominent P2 (the pulmonic component of the second heart sound), and a regular rhythm with no appreciable murmurs. Tachypnea without the use of accessory muscles was noted. Inspiratory crackles were present in the base of both lungs. No wheezing or rhonchi were present. No clubbing, cyanosis, or edema of the arms, hands, legs, or feet was noted. Results of abdominal, neurologic, musculo-skeletal, and cutaneous examinations were normal.

Tani: pulmonary tumor thrombotic microangiopathy

# A rare cause of sudden chest pain and dyspnea: A CARE-compliant case report of Chilaiditi syndrome 9

A 66-year-old woman with cervical squamous cell carcinoma was admitted to the department of gynecology on October 16, 2018. On the second day after the operation, the patient had no abdominal distension and was asked to get out of bed as a preventive measure against thrombosis. At 19:00 hours on the same day, the patient developed acute right chest pain and dyspnea; there was no nausea or vomiting. Her percutaneous oxygen saturation showed a progressive decrease with a minimum level of 75%. Chest auscultation revealed considerable attenuation of respiratory sounds.  **No tachycardia**

Tani: Chilaiditi syndrome

# Unusual Presentation of Multiple Myeloma 10

A 63-year old woman presented at our clinic in a teaching hospital in Yangsan with a history of recurrent common cold and dyspnea over the previous 6 months. During this period, she visited a local clinic several times due to recurrent cough, sputum, and chilling. For 2 weeks prior to visiting our clinic, she felt that her symptoms worsened and had developed shortness of breath even when walking of less than 50 meters, dizziness, and general weakness. She had been diagnosed to have osteoporosis and PE and DVT. At this presentation, her blood pressure and heart rate were 120/80 mmHg and 78 beats per minute, respectively.

Tani: multiple myeloma

# COVID-19-Related Spontaneous Aortic Arch Thrombus With Delayed Embolization 11

# A 62-year-old man with baseline chronic obstructive pulmonary disease was admitted to the hospital with dyspnea and newly diagnosed COVID-19 infection. He denied any symptoms of hemiparesis, sensory deficit, aphasia, facial droop, abdominal pain, flank pain, or extremity pain. Physical exam revealed grossly normal neurological function and strongly palpable pulses in all 4 extremities. No tachycardia

CT: thrombus of the inner curvature of the aortic arch

# The changing face of cancer treatments 12

A 58-year-old woman presented in October 2015 with respiratory-type symptoms. The patient had a history of metastatic lung cancer and currently on chemotherapy. She has tachycardia.

Tani: cardiac tamponade

1. **Hemoptysis: unilateral pulmonary artery atresia? a case report 13**

We present a 55-year-old male with a history of hyper- tension, dyslipidemia, gastroesophageal reflux disease (GERD), and obstructive sleep apnea who presented to our emergency department (ED) with a 3-day history of recurrent cough with 2–3 tablespoons of hemoptysis per episode, chills, and occasional wheezing. He had presented to our ED several months prior with a simi- lar complaint. During the initial admission, he reported one prior event of hemoptysis in his youth and a CTA was performed which revealed an atretic left pulmonary artery and right-sided aortic arch. However, his symptoms were believed to be related to a possible pneumonia and was started on intravenous antibiotics. Approximately nine months after the initial presentation, the patient began experiencing hemoptysis once again, prompting him to visit an urgent care center and was treated for what was once again believed to be a possible pneumonia. He began to experience increased frequency and larger-volume hemoptysis prompting him to present to our ED once again. No tachycardia

Tani: : unilateral pulmonary artery atresia

# Uncommon cause of respiratory failure due to a bezoar in the hypopharynx: a case report 14

A 57-year-old woman with a history of hypertension, diabetes, major depression, and trichotillomania was admitted to the Emergency Department with the main complaints of dyspnea of exertion, shortness of breath, and generalized weakness that lasted for 3 weeks (Fig. [1](https://www.ncbi.nlm.nih.gov/pmc/articles/PMC8739710/figure/Fig1/)a). She also presented dysphagia relative to solids for the past 2 months and a history of snoring at night and drowsiness during the day. She was obese and had a harsh voice. he vital signs were as follows: HR = 112, RR = 19, BP = 135.87, O_2_sat = 75% with FiO_2_ = 21% and O_2_sat = 89% with FiO_2_ = 50%.

Tani: bezoar in the hypopharynx

# Cardiac amyloidosis presenting with coronary artery embolization 15

A 57-year-old female, with a history of hypertension (treated with angiotensin II receptor blockers) and cigarette smoking (35 pack-years), presented to the emergency department, complaining of chest pain with concomitant shortness of breath that started approximately 2 hours before admission. Moreover, the patient reported exertional, gradually deteriorating dyspnoea, starting approximately 6 months ago, accompanied by infrequent episodes of haemoptysis. Three months prior to admission, the patient had undergone a cardiac (clinical examination, electrocardiogram (ECG), transthoracic echocardiogram (TTE)) and pulmonary work-up (clinical examination, spirometry, chest radiography) by her family doctor (as an outpatient), that was reported to be without major pathologic findings and was also scheduled to undergo bronchoscopy. She had a blood pressure of 110/80 mmHg, a heart rate of 97 beats/min and a peripheral oxygen saturation of 93%. Auscultation of the heart and lungs revealed an S4, grade 1 murmurs in the mitral and tricuspid valves and bilateral mild attenuation of breath sounds.

Tani: Heart failure due to cardiac amyloidosis

1. **Hamman-Rich syndrome: a forgotten entity 16**

A 76-year-old male presented to the emergency department with a three-week history of persistent productive cough and shortness of breath. After clinical assessment in the emergency department, a di-agnosis of community acquired pneumonia was established Background history was notable for diabetes mellitus,ischemic heart disease with one previous stent, atrial fibrillation with prior ablation and long-term amiodarone treatment, and a 30-pack year smoking history with no previous record of lung disease. He was prescribed a standard treatment of amoxicillin/clavulanic acid and No improvement was noted on antibiotic therapy and he returned to the emergency department six days later complaining of increasing dyspnoea. Spo2:78. No tachycardia.

Tani: Hamman-Rich syndrome. Acute interstitial pneumonitis

# Postcardiac injury syndrome caused by radiofrequency catheter ablation of persistent atrial fibrillation: severe pulmonary arterial hypertension with severe tricuspid regurgitation: a rare case report and literature review 19

A 70-year-old male was diagnosed with persistent atrial fibrillation. The patient received radiofrequency catheter ablation due to his atrial fibrillation being refractory to antiarrhythmic drugs. The patient was discharged in sinus rhythm (SR). After 3 days, he was admitted to the hospital for dyspnea which gradually worsened, but he had no fever. His heart rate was below 110 beats per minute. Physical examination showed a pansystolic murmur that was heard in the fourth intercostal space at the left edge of the sternum and decreased breath sounds at the base of the lungs. .

Tani: post cardiac injury syndrome

# Atypical chest pain in a young woman with an interatrial bronchogenic cyst 20

# A 31-year-old woman with no medical history presented to the emergency department (ED) for atypical retrosternal chest pain and palpitations of 3 days’ duration associated with mild dyspnoea. The patient had travelled to Mexico 3 months prior but reported no sick contacts. She was found to be irregularly tachycardic with a heart rate of more than 140 bpm.

Tani: intra-atrial bronchogenic cyst

# Atypical Presentation of Small Lymphocytic Lymphoma with Pericardial Effusion 21

# A 61-year-old woman presented to the emergency department with a three-month history of worsening shortness of breath on exertion, which progressed to shortness of breath at rest. She also complained of night sweats, chills, but no chest pain or weight loss. Her medical history included systemic hypertension, a 10 pack-year tobacco smoking history, and type 2 diabetes mellitus. Physical examination showed that her blood pressure was 104/64 mmHg, with a heart rate of 100 bpm, tachypnea with 30 breaths/min, and an oxygen saturation of 93% on 2L of oxygen administered by nasal cannula. She was afebrile with a temperature of 98.1°F (36.7°C). Her jugular venous pressure was >7 cm of H_2_O, she had tachycardia with mildly muffled and distant heart sounds, but no pericardial friction rub, or peripheral edema. Chest percussion was dull over both lung fields. The hepatojugular reflex was absent.

Tani: lymphoma

# Acquired pulmonary arteriovenous malformation associated with bronchiectasis: a case report 22

# A 74-year-old Japanese woman presented with a 6-month history of gradually worsening dyspnea on exertion, a 10-year history of bronchiectasis, a 4-year history of infection with Mycobacterium avium complex, and pulmonary mucosa-associated lymphoid tissue (MALT) lymphoma in remission. She had no history of recurrent epistaxis, and her family history did not include hereditary hemorrhagic telangiectasia (HHT) or PAVMs. Overall, physical examination findings were unremarkable. Her lungs were clear to auscultation, and clubbing or evidence of telangiectatic lesions on the nasal mucosa or skin was absent.

Tani: Pulmonary arteriovenous malformations

# A Case of COVID-19-Associated Free-Floating Aortic Thrombus Successfully Treated with Thrombectomy 24

The patient was a 61-year-old man with a history of hypertension who presented to the hospital for worsening cough and exertional dyspnea. On admission, he was hypoxic and tachycardic and his polymerase chain reaction testing for SARS-CoV-2 was positive. He rapidly developed acute hypoxic respiratory failure, requiring supplemental oxygen through a high-flow nasal cannula (HFNC).

Tani: covid+ aortic thrombosis

# Mediastinal metastatic melanoma: an unusual case presentation of recurrent melanoma 25

A 50-year-old man presented with dull chest pain, worsening dyspnoea with exertion and a non-productive cough over the last 12 weeks. The patient’s medical history was significant for melanoma diagnosed in 2001. The melanoma was localised to the upper right side of his back and was treated with surgical resection only. Otherwise, the patient’s surgical history and medical history were unremarkable. His family medical history was significant for coronary artery disease in his father but no familial cancers. The patient was a non-smoker. Two months prior to presentation, the patient was seen at an outside hospital with similar complaints. At that time, he was found to have a pericardial effusion on echocardiogram. He underwent pericardiocentesis with removal of 1 L of haemorrhagic fluid. Serological evaluation and fluid cultures remained negative, and the aetiology of his effusion was not determined. CT of the chest at the time was reportedly normal. At the time of presentation, the patient was afebrile and in no apparent distress. He had no reported cervical or axillary lymphadenopathy on physical examination. His oxygen saturation was 95% on room air. No tachycardia was observed

Tani: metastatic melanoma

# Catching the GIST: Massive Gastrointestinal Stromal Tumor Presenting as Acute Dyspnea (26)

A 55-year-old Caucasian woman presented to the emergency department complaining of worsening shortness of breath and constipation over three days. The patient reported her abdomen seemed to be enlarging over the past year despite diet and exercise. She reported symptoms of constipation, anorexia, generalized headache and weakness. She believed these symptoms were due to her history of irritable bowel syndrome. The patient reported a 10 pack-year tobacco smoking history. She denied any family history of cancers. On initial physical exam, the patient was hemodynamically stable with a markedly distended, but non-tender abdomen.

Tani: GI stromal tm GIST

# Case 3/2019 - Young Male with Intense Dyspnea, Pulmonary Infiltrate, Normal Cardiac Area and Obliteration of the Apical Portion of the Left Ventricle (27)

This is the case of a 32 year old young, male patient, smoker, who had hypothyroidism post-treatment with radioactive iodine, who sought medical care due to symptoms of dyspnea and cough for a week; these complaints had been preceded by chest pain at exertion two weeks before. Four days after being discharged from the hospital with a diagnosis of hypertrophic cardiomyopathy, the patient sought emergency medical care for severe dyspnea, even at rest and in decubitus, in addition to productive cough. The physical examination disclosed a heart rate of 101 bpm, blood pressure of 112/70 mmHg, pulmonary auscultation showing diffuse crackling rales, cardiac auscultation and abdomen without alterations, and mild edema of legs and feet, without any suggestive signs of deep venous thrombosis.

Tani: goodpasture syndrome

# Azygos Vein Aneurysm with Thrombosis and Aspergillus fumigatusDiagnosed Using Bronchoscopy: Case Report 28

An 86-year-old female patient entered the emergency department (ED) with moderate dyspnea and chest pain. The patient did not tolerate a decubitus position during examination. Past medical history included arterial hypertension (controlled with losartan 100 mg and atenolol 50 mg) and diabetes mellitus type II (DM) (controlled with metformin 850 mg). She had no previous history of hemoptysis. Glycosylated hemoglobin was 7.8% (range 4.0–6.4%). Further interrogation revealed that the patient had parrots (Psittacidae) as pets for at least 10 years. At the time of admission, the patient was oriented and had a Glasgow score of 15/15. Physical examination showed general pallor and jugular vein engorgement. She had normal S1 and S2 heart sounds, with regular rhythm, although tachycardic. Vesicular murmur was abolished in both bases. While in the ED, her condition declined with worsening acute respiratory failure and new-onset paresthesias. She underwent endotracheal intubation and was transferred to the intensive care unit.

Tani: azygos vein dilatation (vascular defects)

# Case report of isolated congenital absence of right pulmonary artery with collaterals from coronary circulation(29)

# A 46-year-old hypertensive female was admitted for evaluation of progressively worsening dyspnea for last 4 years. She was morbidly obese with BMI of 37 kg/m^2^. Her baseline vitals were stable and her O_2_ saturation was 93% at room air.

Tani: absence of right pulmonary artery

# Dual Coronary-Pulmonary Artery Fistula in a Patient with Severe Bicuspid Aortic Valve Stenosis (30)

# A 62-year-old man with no previously established care presented to the emergency department with complaints of fever, productive cough, left lower chest pain, and shortness of breath. He reported exertional chest pain and dyspnea that had progressively worsened before this acute episode. Physical examination findings were as follows: blood pressure 111/77 mm Hg; heart rate 107 beats/minute; respiratory rate 20 breaths/minute; temperature 38.2°C; oxygen saturation 94% on 2 L O2; and a 3/6 harsh late-peaking systolic ejection murmur was heard across the precordium. Bilateral rhonchi were present. Mild bilateral leg swelling was noted. He had no past medical history of heart or lung disease or other known comorbidities. His family history was also negative for known heart disease.

Tani: acute bronchitis+ aortic stenosis+ bicuspid aorta

# Unusual cause of upper gastrointestinal bleed, when OGD could be fatal (31)

# A 57-year-old man with history of hypertension, dyslipidaemia, obstructive sleep apnoea and atrial fibrillation, on rivaroxaban (a direct factor Xa inhibitor), presented to an outside hospital with sudden onset of chest pain, dyspnoea on exertion and one episode of coffee grounds emesis. his wife revealed that the patient had radiofrequency ablation (RFA) for atrial fibrillation 18 days earlier. He was haemodynamically stable on admission. He suddenly experienced an episode of severe chest pain and became unresponsive. He developed wide complex tachycardia and advanced cardiac life support (ACLS) protocol was undertaken. After stabilisation, nasogastric aspiration revealed 300 mL of bright red blood.

Tani: atrial-esophageal fistula

# Primary leiomyosarcoma of the atrium with heterologous differentiation

# (32)

# A 47-year-old woman known to have hypertension, type 2 diabetes mellitus and subclinical hypothyroidism presented with a two-week history of progressive shortness of breath at rest (New York Heart Association Class 4), which was associated with cough, palpitations and mild lower limb edema. Heart rate is 110 bpm.

Tani: leiomyosarcoma of the atrium

# Shortness of breath due to portopulmonary hypertension and hepatopulmonary syndrome: diagnostic challenges and complex management approach in frail patients (33)

# A 60-year-old woman with multiple comorbidities presented to the emergency department with worsening shortness of breath, tiredness and fatigue. She had a background of non-alcoholic fatty liver disease (NAFLD), cirrhosis, type 2 diabetes mellitus (T2DM), atrial fibrillation, ischaemic heart disease, asthma, hypertension, stable mild cerebrovascular accident, hypertension, chronic kidney disease, possible undiagnosed chronic obstructive pulmonary disease (COPD; 30-pack-year history) and severe frailty (Rockwood–Dalhousie frailty score 6). She lived at home with her family, mostly housebound, and required a chair lift to go to the bedroom upstairs. On examination, she was found to have bilateral pitting pedal oedema extending to the mid-thigh. Her oxygen saturation was 92% on air when lying down which dropped to 83% on air when sat upright or trying to mobilise, and was accompanied with an increase in the respiratory rate. Cardiology examination revealed an ejection systolic murmur in the aortic area, radiating to the carotids (suggesting aortic stenosis), a split-second heart sound with a loud second heart sound in the pulmonary area (suggesting pulmonary hypertension) and jugular venous extension along the sternal angle. Her abdominal examination revealed hepatomegaly with an irregular margin, and a palpable spleen with shifting dullness.

Tani: portopulmonary hypertension

# Severe functional mitral stenosis due to a left atrial myxoma masquerading as asthma (35)

# A 45-year-old woman with history of obesity, deep vein thrombosis, pulmonary embolism (PE) and reported asthma presented with recurrent acute on chronic shortness of breath and associated fatigue that were refractory to her usual asthma treatment. Further inquiry highlighted history of chronic dry cough, palpitations, orthopnoea, paroxysmal nocturnal dyspnoea and marked dyspnoea on exertion that significantly limited her functional capacity, with progression over the past year. She denied lower extremity swelling, pleuritic chest pain, sick contacts, fevers, night sweats, significant weight loss or specific triggers for her exacerbations. Physical examination was notable for mild sinus tachycardia and ambulatory desaturation. Pulmonary evaluation demonstrated symmetric chest rise, bibasilar fine crackles, without wheezing. Cardiac evaluation revealed no parasternal heave or displaced maximal impulse, a normal S1 and S2, a third heart sound initially thought to be a ‘fixed split S2’ and later considered as the ‘tumour plop’, and a low-grade diastolic murmur.

Tani: left atrial myxoma

# Marked hemopneumopericardium in a patient with rectal cancer with distant metastasis: a case report (36)

# A 47-year-old Arabic woman, previously diagnosed with rectal cancer metastasized to bones, lymph nodes, and lungs (stage T4N2M1) and post-Hartmann procedure, reported to the emergency department complaining of worsening dyspnea for 2 weeks, significantly more in the supine position. the patient was previously diagnosed with pericardial effusion. There was no reported family or psychological history or any history of other medical illness, but the patient had a productive cough with yellow sputum. On clinical examination, the patient had tachypnea with a respiratory rate reaching 30, a pulse rate of 130 beats per minute, and a temperature of 37 ℃. Oxygen saturation was 95% in the O_2_ mask, she was in 2 L of oxygen, and blood pressure was within normal limits. The patient’s respiratory system examination revealed dullness associated with reduced breath sound on the left side. The rest of the physical tests were unremarkable.

Tani: hemopneumopericardium

# Exertional hypoxia in a healthy adult: a pulmonary arteriovenous malformation (38)

The patient is a 37-year-old Caucasian, active duty US Marine, with a medical history of right shoulder rotator cuff tear with surgical repair, presenting with several months of cough, atypical chest pain, feelings of anxiety and two prior presyncopal episodes with negative neurocardiovascular workup. On presentation, the patient was in no acute distress and haemodynamically stable, with a oxygen saturation of 91%–92%. Physical examination was benign, with no signs of cyanosis and normal pulmonary and cardiac findings.

CT: pulmonary AV malformation

# Pembrolizumab-induced obstructive bronchiolitis in a patient with stage IV non-small-cell lung cancer (39)

A 69-year-old woman with stage iv right upper lobe squamous cell carcinoma with right paratracheal adenopathy and bilateral adrenal and right frontal cerebral metastasis presented to the emergency room for increasing dyspnea for a month and hypoxemic respiratory failure with a saturation of 90% at ambient air. She had received 4 cycles of carboplatin–gemcitabine as first-line chemotherapy and was currently receiving her 7th cycle of pembrolizumab, with an excellent partial tumoural response. Her tumour PD-L1 expression level was 40%. Prior history included pulmonary embolism, for which she was taking rivaroxaban. She was a former smoker and had stopped in 2011. No tachycardia.

1. Tani: obstructive bronchiolitis.

# Rare case of right ventricular dilatation associated with anomalous pulmonary venous drainage, sinus venosus atrial septal defect and persistent left superior vena cava (40)

A patient in her early 37 year old, with a background of asthma, irritable bowel syndrome, fibromyalgia, acne, emotionally unstable personality disorder, anxiety (with panic attacks) and depression, presented to the emergency department with shortness of breath, cough and pleuritic-sounding left-sided chest pain. Over the preceding 3 months, she had contacted the general practice out-of-hours service and her general practitioner (GP) with similar symptoms. She was managed with steroids and antibiotics as an infective exacerbation of asthma. After her second presentation she underwent an outpatient chest X-ray, which led to a cardiology referral for cardiomegaly after further questioning by her GP revealed a 3-month history of palpitations. Her regular medications included aripiprazole, mirtazapine, Duoresp and Ventolin inhalers, cetirizine, promethazine hydrochloride and combined contraceptive pill. She had a family history significant for two paternal relatives with valvular problems and a sudden death of her paternal uncle at the age of 40. On general examination, she was visibly anxious and tachypnoeic with a respiratory rate of 24. She was not requiring oxygen, with saturation of 96% on room air, and auscultation of the chest revealed no abnormalities. She was normotensive with a blood pressure of 109/73 mm Hg and a sinus tachycardia with a heart rate of 109 beats per minute. Her jugular venous pressure was not raised, and she had no peripheral oedema.

Tani: pulmonary arterial pressure

# Platypnea-orthodeoxia Syndrome Induced by an Infected Giant Hepatic Cyst (41)

An 83-year-old man was admitted to our hospital due to the exacerbation of dyspnea without any other respiratory symptoms. He denied a history of chest pains, palpitations, dizziness and hematochezia. Over the five years preceding this admission, he had experienced recurrent episodes of oxygen desaturation, with oxygen saturation levels less than 85% in the upright position that improved to more than 90% in the recumbent position.

The patient had a history of a colonic arteriovenous malformation, hypertension, hyperuricemia and a hemorrhagic gastric ulcer. He took febuxostat 10 mg and lansoprazole 15 mg both once daily. He was a non-smoker and consumed no alcohol. There was no history of any drug or food allergies. His family history and social history were otherwise non-contributory.

On a physical examination, he appeared mildly unwell. He was afebrile with a tachycardia of 107 beats per minute, a blood pressure of 159/99 mmHg, a body temperature of 37℃ and tachypnea with a respiratory rate of 24 breaths per minute in the recumbent position. Pulse oximetry on continuous oxygen therapy at a rate of 10 L/min demonstrated an oxygen saturation above 90% in the recumbent position, which dropped to less than 80% when sitting upright. No conjunctival pallor, icterus, cyanosis, digital clubbing or spider nevi were detectable on the physical examination. A cardiovascular examination revealed a normal jugular venous pressure and heart sounds with no detectable murmurs. A respiratory examination revealed a short trachea and diminished breath sounds in the right lung base. The abdomen was non-distended and without scars. The liver span was approximately 10 cm with no detectable ascites. The liver was palpable 3 cm below the costal margin without tenderness. There were no peripheral stigmata of systemic diseases. Examinations of the genitourinary, neurological, dermatological and musculoskeletal systems were all normal.

Tani: Platypnea-orthodeoxia Syndrome

# Use of awake proning to avoid invasive ventilation in a patient with severe COVID-19 pneumonitis 42

# A 60-year-old Asian male non-smoker with no significant medical background presented to our accident and emergency (AE) department with significant and worsening shortness of breath. He had been self-isolating with COVID-19 symptoms and was desaturating to SpO_2_ 88% on arrival in AE. He initially required low flows of oxygen (<4 L) to maintain his saturations above 92%. Covid test came in positive. The following morning, the patient became more hypoxic

Tani: covid pneumonitis

# Coexisting pulmonary haemorrhage and venous thrombosis: a tricky but novel case (43)

A woman aged 43 years with no significant medical history presented with a 7-day history of worsening dysphagia, non-productive cough, hoarseness, fever, rigors, general malaise and anorexia. She was a never smoker and was on no regular medication but had completed a recent course of clarithromycin. At presentation, her cough had become productive of foul-smelling brown sputum. On examination, the patient was tachypnoeic and tachycardic without haemodynamic compromise. She was feverish at 38.5°C and oxygen saturations were 94% on air. Chest auscultation revealed reduced air entry with crackles at the left lung base. Her tonsils were erythematous and there was right-sided cervical lymphadenopathy.

Tani: lemierre’s syndrome

# Spontaneous giant pseudoaneurysm in the upper segment of the aortic arch (44)

A 57-year-old male patient was referred to the Department of Emergency with a chief complaint of a 2-month history of back pain and short of breath for 1 day. Because the patient lived in a remote area, he did not seek any form of regular treatment during the 2 months. Meanwhile, he developed hypertension 1 month ago that was not treated. The patient denied any history of trauma, operation, allergy, any types of hepatitis, or other potential infectious diseases.

Tani: Aortic aneurism

1. **A 44-Year-Old Woman with Dyspnea and Hemoptysis in the Setting of Remote Bariatric Surgery**

A 44-year-old woman was transferred to the ED from an outside hospital because of hemoptysis and concern for left-sided pulmonary infiltrate with associated pleural effusion. On arrival to the ED, she was in acute hypoxic respiratory failure. The patient was intubated emergently and was admitted to the medical critical care unit for further treatment. On presentation, the patient’s vital signs revealed a temperature of 36.8 C, a heart rate of 161 beats/min, BP of 137/61 mm Hg, and a respiratory rate of 65 respirations per minute with oxygen saturation of 97% on 6 L of oxygen by nasal cannula. Before intubation, the patient was noted to have conversational dyspnea and was actively coughing up maroon-colored sputum. She was alert and able to answer questions. Dry, brown blood was observed in the oropharynx. She denied any chest or abdominal pain. Diminished breath sounds were noted bilaterally without wheezes or crackles. No rashes, oral lesions, or obvious joint swelling or deformity was noted. The remainder of her physical examination was unremarkable. Batient has a history of bariatric surgery

Tani: Gastropulmonary fistula resulting in necrotizing pneumonia

# Takotsubo syndrome as an overlooked and elusive cause of a single episode of dyspnea in young women: a case report

A 23-year old woman with no underlying chronic disease underwent closed reduction surgery for a nasal bone fracture under general anesthesia (with sevoflurane as the anesthetic). Approximately 5 h later, she presented to the emergency department with dyspnea Her dyspnea improved upon arrival; she was, otherwise, symptom free. Her vitals upon arrival were stable: oxygen saturation, 100%; blood pressure, 121/83 mmHg; pulse rate, 105 beats/min; and respiratory rate, 16 breaths/min. Exactly 4 h and 40 min prior to the onset of symptoms, she had undergone a closed reduction surgery for a nasal bone fracture under general anesthesia. The course of surgery was uneventful, and the patient was discharged after observation as she showed no unusual signs postoperatively. After arriving home, she was drinking a glass of water when the dyspnea started. This symptom lasted 30 min. She had no history of a recent upper respiratory infection or immobilization, did not smoke or drink alcohol, and had no family history of cardiac or cerebrovascular diseases. The physical examination showed no signs of neck vein engorgement or leg edema; there were also no unusual results pertaining to the lung, heart, or abdomen.

Tani: Takotsubo syndrome

# Botulism presenting as dyspnea and respiratory failure in the Canadian Arctic

A 58-year-old Inuvialuit woman presented to her health centre in the western Canadian Arctic with acute-onset (over one day) dyspnea without chest pain or fever. She had tachypnea with increased work of breathing. A chest radiograph appeared normal. The patient soon had a decreased level of consciousness and required positive pressure ventilation to maintain adequate oxygenation and ventilation. She remained hemodynamically stable. An airplane ambulance was flown to her community, and the patient was intubated by the paramedics. The neurologic examination was notable for bilaterally dilated (6 mm) pupils; findings were otherwise normal, including strength and deep tendon reflexes. Obtaining a detailed history from the patient in this context was challenging. Her husband reported that she had complained of dry mouth and mild nausea one day before her breathing difficulties. She had a smoking history of 40 pack-years, with no important medical history or medications. No one in her family or community reported being ill.

Tani: botulism

# What is Your Call (49)

A 54-year-old woman presented to the emergency department with increasing shortness of breath over four days. She reported an increased cough over the previous two months and that her daughter recently had an upper respiratory tract infection. The patient had metastatic breast cancer (estrogen-receptor positive, human epidermal growth factor receptor 2/neu negative). She had received first-line paclitaxel chemotherapy four years earlier, but severe neuropathy developed; her regimen was changed to doxorubicin weekly, with good response and a maximum lifetime cumulative dose of 450 mg/m^2^. Restaging performed three months before this visit showed progression in the liver, resulting in a new regimen that included everolimus and exemestane. The patient also had a brief history of treated hypertension a few years earlier but did not take any other medications. Review of systems was negative for fever, pleuritic chest pain and asymmetric calf tenderness. In the emergency department, the patient’s blood pressure was 130/90 mm Hg, heart rate was 86 beats/min, oxygen saturation was 97% on room air and oral temperature was 36.6°C. Her jugular venous pressure was difficult to assess. Precordial examination was unremarkable. She had decreased breath sounds at the bases bilaterally on respiratory examination. Abdominal examination was normal. She had no evidence of peripheral edema or clubbing.

Tani: congestive heart failure

# Uncommon presentation of COVID-19: Gastrointestinal bleeding (50)

# A 53 year old male patient with a medical history of hypertension, diabetes mellitus, and chronic renal failure presented to emergency department with epigastric pain, diarrhea, and dyspnea. The patients stated that he presented to the emergency department due to dyspnea and epigastric pain two days before this application, and a nasal swab was taken for COVID-19, and symptomatic treatment was initiated. The patient presented to the emergency department again since his complaints did not regress, and he also had diarrhea started. The patient stated that he had no fever or cough. He had dyspnea, palpitation, and black stool. He had no recent travel outside of the state or internationally, no contact with COVID-19 (+) patient. He had no drug usage in his history except for the use of acetylsalicylic acid. The vital signs were blood pressure: 130/100 mmHg, pulse: 108 beats.min^−1^, O_2_ saturation: 99%, respiration rate: 18 min^−1^, Glasgow Coma Score: 15 (G4M6S5). The heart was rhythmic and tachycardic. Covid test +.  No abnormality was found on the patient's cardiopulmonary or respiratory examination. He had epigastric tenderness in the abdominal examination. Melena was present in his rectal examination.

Tani: gis kanama

# Hereditary haemorrhagic telangiectasia and pulmonary arteriovenous malformations (51)

# A 33-year-old female UK resident presented to the emergency department with a 3-month history of increased lethargy and shortness of breath on exertion during the COVID-19 pandemic. She had no history of coryzal symptoms, dysphagia, dysphonia, otalgia or weight loss. Her SpO_2_ was recorded at 88% on air on exertion at home. There was no history of chest pain. Her medical history included migraine and epistaxis. She was recently diagnosed with hyperthyroidism secondary to Graves’ disease. Her medications included carbimazole 20mgs and sumatriptan 50mgs. She had no allergies. Her surgical history was unremarkable. Two years previously, her son, born by normal vaginal delivery, sustained an intracerebral haemorrhage aged 4 days thought likely secondary to a vascular malformation. She was a non-smoker, did not consume alcohol and was a keen runner. She was tachycardic, hypertensive and hypoxic with observations as follows: heart rate 125 beats per minute, BP 153/95 mm Hg, SpO_2_ 88% on air, respiratory rate 22 and temperature 37°C. Neck examination confirmed a lump in the lower part of the right lobe of her thyroid gland. Oral mucosal examination demonstrated telangiectasia. Physical examination showed normal heart sounds. She was warm and well perfused with a capillary refill time of 2 s. Her jugular venous pulse was not raised. Her abdomen was soft and non-tender. Bowel sounds were audible on auscultation. Respiratory examination confirmed good air entry bilaterally. A bruit was audible at the lower left lung base. Skin examination was significant for macular, blanching telangiectasias on her fingers, lips and ears.

Tani: Pulmonary artery malformations

# Cardiac MRI-confirmed mesalamine-induced myocarditis (52)

A 38-year-old Caucasian man presented to the emergency department (ED) with stabbing, pleuritic, substernal chest pain over the previous 2 days. The patient's chest pain was worsened by lying down and was accompanied by nausea, vomiting, dyspnoea and a fever of 104.4°F. His medical history was significant for IBD diagnosed following a flexible sigmoidoscopy ∼3 weeks prior (later diagnosed as Crohn's disease); on diagnosis of IBD, he was started on mesalamine delayed-release capsules (Delzicol) 800 mg three times/day (2.4 g/day) and a mesalamine suppository (Canasa) 1000 mg at bedtime. Despite 1 week on this regimen, the patient continued to have bloody diarrhoea two to three times/day and his oral mesalamine was increased to 2.4 g twice/day (4.8 g/day). One-week later, he developed multiple aphthous ulcers in his mouth and throat and was started on prednisone 40 mg daily to be slowly tapered over 1 month (prednisone was 20 mg once daily on admission). Other home medications included escitalopram 20 mg once daily for anxiety. The patient had no prior surgeries, was a non-smoker and was abstinent from alcohol for the previous month. The patient had no family history of premature coronary artery disease or sudden cardiac death. ital signs in the ED were heart rate (HR) 127 bpm, blood pressure 101/64 mm Hg, respiratory rate 18 per minute, oxygen saturation 94% breathing room air and temperature 99.2°F. Physical examination revealed laboured breathing and normally split S1 and S2 heart sounds with no murmurs, rubs or gallops.

Tani: myocarditis

# Treatment of Hypertensive Cardiogenic Edema with Intravenous High-Dose Nitroglycerin in a Patient Presenting with Signs of Respiratory Failure: A Case Report and Review of the Literature (53)

# A 63-year-old male Hispanic patient presented to hospital as an emergency with sudden onset of severe respiratory distress, palpitations, and profuse sweating. He was in his usual state of health and could feely ambulate with independent activities of daily living until 30 minutes before arrival to emergency room. He had a 20-year history of hypertension, a 15-year history of diabetes mellitus, coronary artery disease requiring insertion of a coronary artery stent five years previously, a ten-year history of New York Heart Association (NYHA) Class II heart failure with a reduced ejection fraction (EF) of 35%, chronic kidney disease (CKD) stage 3B. On examination, his blood pressure was 205/110 mmHg, his respiration rate was 29 breaths/min, his heart rate was 118 beats/min, pulse oximetry was 82% (in room air), and his temperature was 36.2°C. His weight was 72 kg and his height was 68 inches, resulting in a body mass index (BMI) 24.13 kg/m^2^. On physical examination, the patient was in severe respiratory distress, he was sweating, and unable to speak clearly. His jugular venous pressure was elevated, as shown by jugular vein distention (3+). On chest auscultation, profuse bilateral crackles were present in all pulmonary fields. Cardiac auscultation was remarkable for a gallop with a regular rate along and a systolic murmur 3/6 heard at the left midclavicular line, at the level of the fifth intercostal space.

Tani: Hypertensive Cardiogenic Edema

# An Atypical Case of Silent Aortic Dissection in a Peritoneal Dialysis Patient: A Case Report and Review of Literature (54)

A 53-year-old African American male with a history of human immunodeficiency virus infection (HIV) controlled on treatment, chronic kidney disease stage5d (CKD5d) on PD since 2016 and renal transplant waitlist, uncontrolled hypertension, obstructive sleep apnea, and non-ischemic cardiomyopathy (ejection fraction [EF] of 50–55% on transthoracic echocardiography and EF of 38% on nuclear stress test) presented to the Emergency Room with worsening orthopnea and exertional dyspnea for 3 days. His initial examination was significant for blood pressure (BP) 140/80 mm Hg, heart rate100 bpm, temperature 36.7°C (98.0°F), respiratory rate 18 breaths/minute and O_2_ saturation 98% on room air. Lung auscultation revealed bilateral rales. he rest of his physical examination was within the reasonable limits.

Tani: Aortic dissection

# Platypnoea–orthodeoxia after left total knee replacement

An 82-year-old woman was electively admitted for left TKR. Her medical history included acute myocardial infarction 10 years prior, as well as hypertension, left total hip replacement and a stable, 4.5 cm ascending aorta aneurysm. She was taking the following medications: telmisartan 40 mg once a day, aspirin 100 mg once a day, metoprolol 50 mg two times per day, rosuvastatin 10 mg once a day, rabeprazole 20 mg and lercanidipine 10 mg. Following surgery, the patient complained of increasing shortness of breath and was noted to be hypoxic on room air, up to 86%. A physical examination did not reveal any other significant findings.

Tani: Platypnoea–orthodeoxia syndrome

# Systemic sclerosis: severe pulmonary arterial hypertension and pericardial effusion at diagnosis (56)

# A 51-year-old female smoker with a history of arterial hypertension and depression presented to the Emergency Room (ER) with complaints of fatigue for the past 1 month. She also mentioned a respiratory tract infection characterised by rhinorrhoea, non-productive cough and myalgia, weeks before the present clinical picture. Physical examination revealed high blood pressure (blood pressure 171/116 mm Hg) with peripheral capillary oxygen saturation of 94% and a systolic heart murmur. No tachcardia.

CT: pericardial effusion and emphysema

# Para-aortic lymphadenopathy associated with adult COVID-19 multisystem inflammatory syndrome

A 21-year-old Caucasian man arrived at the hospital with dyspnoea and abdominal pain after a 5-day history of influenza-like symptoms: cough, headache, fever and sore throat. The patient had not received any medication other than analgesics. In the emergency department, a nasopharyngeal real-time reverse transcription PCR (RT-PCR) test was performed, which was positive for SARS-CoV-2. She was diaphoretic, with a blood pressure of 70/50 mm Hg, a heart rate of 100 beats/min, a respiratory rate of 40 breaths/min and oxygen saturation of 88% on room air. On physical examination, there were bilateral crackles in the lungs with a visible jugular venous pulse. On cardiac auscultation, there was only tachycardia, and her abdomen was tender on palpation, without signs of peritonitis. Hepatosplenomegaly was not present.

Tani: covid pneumonitis

# A 77-Year-Old Woman With Capillary Hypoxia and Perioral Cyanosis (58)

A 77-year-old woman with asthma, hypothyroidism, irritable bowel syndrome, overactive bladder, and multiple rheumatologic conditions was sent from the clinic to the ED for evaluation of hypoxia. In the clinic, she reported dizziness without shortness of breath and was noted to have perioral cyanosis with an oxygen saturation measured by pulse oximetry (Spo_2_) of 80%. She was given a nonrebreather mask delivering oxygen at 8 L/min, but the Spo_2_ remained at 77% to 82%. In the ED, the patient reported intermittent shortness of breath, 2 to 3 days of mild left lower extremity swelling, and a brief episode of lightheadedness earlier in the day that had since resolved. She denied fevers/chills, upper respiratory symptoms, and chest pain. She had been referred to the pulmonology clinic 3 years earlier to evaluate mild hypoxia with Spo_2_ readings in the low 90% range, but pulmonary function testing failed to identify an etiology. There was no history of VTE. Her rheumatologic conditions included osteoarthritis, rheumatoid arthritis, Sjögren’s syndrome, and fibromyalgia. On arrival to the ED, the patient’s initial vital signs revealed a heart rate of 70 beats/min; BP, 150/79 mm Hg; respiratory rate, 18 breaths/min; and an Spo_2_ of 81% while receiving oxygen through a nonrebreather mask at 15 L/min. On initial examination by the medical ICU team, she was again noted to have perioral cyanosis and a grayish hue to her fingertips without clubbing. Pulmonary examination, completed with the patient received oxygen via a high-flow nasal cannula at 60 L/min with an Fio_2_ of 100%, revealed a respiratory rate of 16 breaths/min and symmetric vesicular breath sounds bilaterally without any adventitious breath sounds. Her Spo_2_ remained consistently in the mid-80% range during the interview and examination, and she was not in distress. She had minimal lower extremity edema, with the left lower extremity appearing slightly larger than the right lower extremity. The remainder of the examination yielded unremarkable results. the patient also reported current use of duloxetine and ginkgo biloba

Tani: Sulfhemoglobinemia,

# Superior Vena Cava Syndrome in Conjunction with Pulmonary Vasculature Compromise: A Case Study and Literature Review (61)

# Superior Vena Cava Syndrome in Conjunction with Pulmonary Vasculature Compromise: A Case Study and Literature Review (61)

A 61-year-old Caucasian male with a history of heavy tobacco use for many decades presented to the Emergency Department because of significant dyspnea at rest associated with swelling in both of his lower extremities. His examination revealed congested neck veins, normal breath sounds, and a regular pulse. He required 3 L of oxygen via nasal cannula to maintain an oxygen saturation of 97%.

Tani: SVC syndrome

# Isolated right ventricular infarction: a diagnostic challenge (62)

We present a case of a 73-year-old woman admitted to the emergency room due to sudden-onset dyspnoea, prostration and slowed speech. The beginning of dyspnoea had occurred 4 h earlier and was accompanied by diaphoresis and malaise. At admission, the patient was hypotensive (70/30 mm Hg) and bradycardic, with signs of poor peripheral perfusion. Her body temperature was normal, she had a slightly increased respiratory rate and saturation of peripheral oxygen was 92%. Jugular distention was visible but there was no paradoxical pulse. No changes in heart sounds and no lung auscultation were noted. There was neither symmetrical nor asymmetrical oedema of lower extremities. Despite being prostrate, the patient remained oriented and answered questions with slowed speech (Glasgow Coma Scale (GCS)=13). Her medical history included hypertension and an ischaemic stroke 8 years prior, with consequential dysarthria and chronic use of carvedilol 6.25 mg two times a day, telmisartan 40 mg four times a day, amlodipine 5 mg four times a day, furosemide 40 mg four times a day and clonidine 0.15 mg four times a day. mplaints: Dyspnea, prostration, slowed speech , diaphoresis

Tani: Isolated right ventricular infarction

# Entrectinib-related myocarditis in a young female patient with metastatic non-small cell lung cancer (case 63)

A 51-year-old woman with a recent diagnosis of stage IV non-small cell lung cancer (NSCLC) initiated treatment with entrectinib 600 mg orally once daily. Two weeks after, she was referred to the emergency department for observation from an outpatient cancer centre due to sudden onset of pressure-like chest pain that irradiated to the interscapular region and dyspnoea. She had no known cardiovascular risk factors. The pain appeared at rest while she was in bed, was not aggravated by movement nor inspiration and was associated with shortness of breath and a feeling of being unwell. Before admission to the emergency department, she self-medicated with non-steroidal anti-inflammatory drugs, which seemed to alleviate the pain. Review of systems was negative for orthopnoea, peripheral oedema, dizziness or palpitations. On clinical examination, she was hypotensive with a blood pressure of 86/54 mm Hg, heart rate of 75 bpm, respiratory rate of 18 breaths/min and oxygen saturation of 94% on room air. She was afebrile with a temperature of 37.4°C. Physical examination was unremarkable.

Tani: myocarditis

# Total electrical alternans in a patient with malignant pericardial tamponade (64)

# We present a case of a 59-year-old man, with a medical history of marijuana dependence and schizophrenia. His regular medication included penfluridol, and he was a heavy smoker (over 50 pack years). He was presented to our hospital emergency room with colicky abdominal pain in the right upper quadrant and epigastric area with urge to move, nausea, vomiting, decreased appetite and increasing shortness of breath for the past 2 days. On examination, the patient was tachypnoeic (with a frequency of 28 per minute), peripheral oxygen saturation was 91% with 15 L of O_2_ via a non-rebreather mask, and he had an increased heart rate of 110 beats per minute. There was no sign of hypotension, however, a narrow pulse pressure (128/103 mm Hg) and elevated jugular venous pressure were observed. His heart sounds were classified as normal. No pulsus paradoxus was reported. The patient had a maximum score on the Glasgow coma scale and was oriented in time, place and person. He had a normal temperature (37.1°C) and his glucose level was 8.6 mmol/L. He had crackles at the base of the right lung and palpation of the right upper quadrant of the abdomen revealed direct tenderness.

Tani: lung carcinoma, cardiac tamponade , pleural effusion

# Platypnea Orthodeoxia Due to a Patent Foramen Ovale and Intrapulmonary Shunting After Severe COVID-19 Pneumonia

# An 85-year-old man (BMI 21.24 kg/m^2^) with a past medical history of hypothyroidism, hyperlipidemia, and type 2 diabetes mellitus presented to the Emergency Department after a fall and was found to be in hypertensive emergency with a left temporal lobe hemorrhage. The patient was febrile to 38.4°C, slightly tachycardic at 101 beats per minute, and hypertensive at 186/94 mmHg. At that time, the patient had no subjective symptoms of respiratory distress but was found to be hypoxic with an oxygen saturation of 91% on ambient air. He received oxygen supplementation by nasal cannula (NC), with improvement in his oxygen saturation. The initial physical exam was unrevealing. COVID-19 was suspected and PCR testing was positive for the same. On hospital day 3, the patient began to experience dyspnea associated with desaturations on pulse oximeter as low as 70%.  In the subsequent days, his oxygen requirement slowly decreased, He was discharged to a skilled nursing facility. He was discharged to a skilled nursing facility (SNF) on 4 L by NC after 15 days of hospitalization, when his condition appeared to plateau in terms of his oxygen requirement and management of his medical co-morbidities. Two days after discharge and approximately day 17 after diagnosis, he re-presented to the Emergency Department after an oxygen desaturation to 77% on 6 L by NC. A physical exam demonstrated bilateral wheezing and rhonchi. He required high-flow oxygen at 60% and 25L to improve his O2 saturation to 92%.

Tani: platypnea orthodeoxia

# Acute respiratory failure as initial manifestation of conventional osteosarcoma rich in giant cells: a case report

# The patient was a 25-year-old Hispanic woman of African descent. She was the youngest of four siblings, a nursing student, and worked part-time in the administrative department of the Cali City public transportation system. She was previously healthy, was a non-smoker, and did not consume alcohol or other drugs. Her father had arterial hypertension and her mother had type 2 diabetes. She had no other family history. The patient’s medical history included a 1-month history of dyspnea on exertion, intermittent dry cough, hyporexia, and intermittent unquantified fever. She did not seek medical attention for these symptoms until she presented with a sudden increase in dyspnea, at which time she presented to the emergency department of a peripheral institution. Upon admission, she went into acute respiratory failure and cardiorespiratory arrest, with pulseless electrical activity. Despite orotracheal intubation, the patient was difficult to ventilate. Her vital signs were: blood pressure, 200/140 mmHg; heart rate, 100 beats per minute; respiratory rate, 16 breaths per minute; temperature, 36 °C; oxygen saturation, 78% despite being on ventilatory support. Physical examination revealed pupils with anisocoria (right 5 mm, left 3 mm) and thick neck with jugular engorgement with no masses. There were no skin lesions. The cardiopulmonary evaluation revealed sinus tachycardia, and on auscultation of the lung, bilateral thick rales were found. There was no collateral circulation, ascites, or masses on the abdomen. Extremities were without edema.

Tani: Osteosarcoma

# A rare case of obstructive right atrial lipoma case

# A 71-year-old Caucasian woman presented to the emergency department with acute exacerbation of a 2-year history of recurrent dyspnoea and a new onset manifestation of hypertensive crisis. She did not complain of angina. Her past medical history included essential hypertension, dyslipidaemia, diabetes mellitus type 2 and obesity. Her past surgical history was significant for strumectomy in 1991 and tonsillectomy in early childhood. On physical examination, Kussmaul’s sign was detectable on inspiration, suggestive of an increased jugular venous pressure. No tachycardia.

CT: atrial mass

# A 45-Year-Old Man with Scleroderma Renal Crisis Associated with a History of Systemic Sclerosis Sine Scleroderma

# A 45-year-old man with a past medical history of essential hypertension for 3 years, not requiring any medications, presented to the Emergency Department with worsening nausea and vomiting for 3 weeks. The patient stated feeling nauseous every day, and vomited after drinking juices or sodas. He had been tolerating solid meals without complaints. Additionally, the patient had progressive exertional dyspnea when walking around the house or walking upstairs. He reported orthopnea and bilateral pedal edema. He denied smoking, alcohol use, or illicit drug use. Family history was notable for systemic lupus erythematosus in his aunt, and diabetes in his mother. Initial vital signs showed body temperature 36.6°C, heart rate 95 beats/min, respiratory rate 19 breaths/min, blood pressure 182/108 mmHg, and oxygen saturation 99% on room air. Physical examination was significant for mild jugular venous dilation, fine crackles in both lungs, and 1+ pitting edema bilaterally. The skin appeared normal with modified Rodnan score of zero without nail changes, sclerodactyly, telangiectasia, or Raynaud’s phenomena. He was admitted to the intensive care unit because of hypertensive emergency with AKI . The patient became gradually hypoxic with increased oxygen requirement from room air to 6 liters via nasal cannula.

Tani: systemic sclerosis sine scleroderma (ssSSc) with renal crisis

# Adalimumab as a potential cause of drug-induced thrombocytopaenic microangiopathy

We report the case of a 63-year-old man referring to the emergency department (ED) for confusion, asthenia, dyspnoea on exertion, worsening diarrhoea and peripheral oedemas. He correlated symptoms’ onset with the recent initiation of adalimumab (one single dose) as a new treatment for his Crohn’s disease, 15 days before the admission to our ED. History revealed Crohn’s disease diagnosed 16 years before and complicated with abdominal abscesses which required several surgical interventions of intestinal resection in the previous years. He also complained of hypersensitivity to infliximab and intolerance to azathioprine. At the admission in the ED he was confused, with no other pathological neurological sign; his skin was pale; cardiac tones were regular but tachycardic, with no added bruits; abdomen was tender, with no evocable pain; liver and spleen were not clinically enlarged; he was dyspnoeic, with regular breath murmurs and some rales; we also observed a modest peripheral oedema; arterial blood pressure was 90/60 mm Hg, cardiac frequency was 102 bpmR (beats per minute, rhythmic), respiratory rate was 22 breaths/min, SpO_2_ was 96% while breathing room air.

Tani: drug-induced thrombocytopaenic microangiopathy

# Postnatal diagnosis of maternal congenital heart disease: missed opportunities

A 30-year-old primigravida with no known comorbidities presented to the emergency department at 29+6/40 gestation, with breathlessness. She had no known comorbidities. The patient presented to our district general hospital emergency department at 29+6 weeks gestation. She described a 7-day history of shortness of breath with no chest pain or palpitations. Examination demonstrated tachycardia at 104 bpm, a respiratory rate of 20, oxygen saturations of 93% on air and a blood pressure of 145/86 mm Hg. Auscultation of the patient's chest and her chest X-ray were normal.  The patient remained cardiovascularly stable throughout an uneventful caesarean section. on postop day 1, The patient's oxygen requirements continued to increase

Tani: Eisenmenger’s

# Late presentation of myocardial infarction (MI) during the COVID-19 pandemic

A 65-year-old man with a prior pulmonary embolism (PE) 10 years ago presented to the emergency department with progressively worsening dyspnoea, orthopnoea and leg oedema. Approximately 3 weeks prior to this presentation, he described an episode of crushing, substernal, 10/10 pain radiating to his left flank accompanied by diaphoresis that lasted over 2 days. He thought this presentation was a symptom of COVID-19, so he self-quarantined and suffered through the pain. Given his history of PE, he tried breathing exercises to try to alleviate the pain; however, his breathing continued to worsen to the point of requiring emergency care.

Tani: MI

# Unusual presentation of haemoptysis following accidental inhalation of the ‘superwarfarin’ rodenticide brodifacoum

A 56-year-old Caucasian woman presented to the emergency department with acute onset haemoptysis and dyspnoea, which resolved after three episodes. This haemoptysis was observed by clinicians, and it is estimated that between 250–300 mL of frank blood was expectorated. The patient had no medical history, no known allergies and took no regular medication. There was no relevant family history, and the patient was a never-smoker who reported no history of exposure to tuberculosis or asbestos. She kept no pets and her occupation was office-based. The evening prior to presentation, the patient had entered a confined, unventilated ceiling-space area that contained the rodenticide brodifacoum, which had lain in situ for approximately 2 years. Immediately following this brief exposure, the patient reported a transient dry cough, but no other symptoms. She recalled spending about 4 min in this attic space and noted that the coating of the brodifacoum pellets had degraded, exposing the powdered brodifacoum within. Approximately 6 hours later, she woke in the early morning distressed and acutely dyspnoeic, and coughed up blood. On clinical examination in the emergency department, there were no signs of respiratory failure, with stable oxygen saturations maintained without supplemental oxygen. Cardiovascular examination was noted to be normal and there was no overt evidence of haemorrhage into other sites, nor systemic features suggestive of alternative pathology.

Tani: Haemorrhage secondary to rodenticide exposure

# Pulmonary Tumor Thrombotic Microangiopathy with Administration of Pulmonary Vasodilator Resulting in Clinical Improvement Prior to Final Diagnosis

# An 80-year-old Japanese woman suffered dyspnea and palpitations for several days. Because her symptoms worsened, she was transferred to the emergency room of South Miyagi Medical Center, Miyagi, Japan. She had a history of hypertension and hyperlipidemia, which were under medical control, and breast cancer, which was in complete remission following surgical treatment 30 years earlier. She did not smoke or drink alcohol. Her clinical findings in the emergency room were as follows: blood pressure (BP), 107/55 mmHg; heart rate, 100 beats/min; respiratory rate, 24 breaths/min; and oxygen saturation (SpO2), 85% on 2 L/min of O2. She had no fever, cough, or edema. HR:100 beats per min.

Tani: Pulmonary Tumor Thrombotic Microangiopathy due to gallbladder carcinoma

# A large chronic pericardial effusion in an ultramarathon runner with anti-CCP positive rheumatoid arthritis

# A 40-year-old male extreme endurance runner, diagnosed with anti-CCP positive RA threeyears previously, presented with a six-week history of pleuritic chest pain, exertional dyspnoea and a non-productive cough. The patient had just returned to the UK from the far east after an ultramarathon (typically classified as races over 50 km), where he reported several near-syncopal episodes on exertion and episodes of right-sided pleuritic chest pain. These symptoms occurred acutely on a background of a four-month history of fatigue and weight loss (4 kg). His initial treatment for RA was an intramuscular depot of dexamethasone in addition to disease-modifying antirheumatic drugs (DMARDs) which had recently been changed from methotrexate to sulfasalazine. On observation, the patient was found to be unwell but clinically stable with vital signs within normal ranges. The patient was apyrexial, normoxic and normotensive, with a heart rate of 72 bpm (a relative tachycardia considering the patient’s resting heart rate of 42 bpm). On examination, dullness to percussion was noted at the right lung base. On auscultation, heart sounds were muffled but no pericardial rub was audible. Significant negative findings included a non-raised jugular venous pressure, no pulsus paradoxus and no signs of active inflammatory arthritis. 75

CT: pericardial effusion

# Cardiac Sarcoma: Unusual Cause of Intracardiac Contrast Filling Defect

# A 28-year-old female was transferred to the emergency department from her physician’s office for further evaluation of tachycardia. She was being seen for a recent illness which included nausea, vomiting, diarrhea and fevers. The patient endorsed fatigue, dyspnea on exertion, and extremity edema. She had no chest pain or cough. Exam revealed a pale, fatigued, mildly ill-appearing female with bilateral lower extremity edema and diminished breath sounds on the right.

Tani: Cardiac Sarcoma

1. **Lung cancer mimicking massive pulmonary embolism**

A 65-year-old woman presented to the emergency department with nausea and vomiting after starting erlotinib as second-line treatment for non-small-cell lung cancer (T4N3Mx). She had previously received chemotherapy and radiotherapy. She also had a background of chronic obstructive pulmonary disease. On examination, oxygen saturations were 80% on air, she was febrile at 39.5°C, tachycardic at 139 bpm and had a low normal blood pres- sure of 104/75 mm Hg. She was initially thought to be dehydrated as well as having a lower respiratory tract infection. She was commenced on intravenous antibiotics and fluids. The day following admission, she remained tachycardic and hypotensive despite aggressive fluid resuscitation. She was found to have a persistently raised jugular venous pulse. She became increasingly hypoxic

tani: mediastinal mass
